# Feasibility of monitoring Global Breast Cancer Initiative Framework key performance indicators in 21 Asian National Cancer Centers Alliance member countries

**DOI:** 10.1016/j.eclinm.2023.102365

**Published:** 2023-12-16

**Authors:** Sok King Ong, Rei Haruyama, Cheng Har Yip, Tran Thu Ngan, Jingmei Li, Daphne Lai, Yawei Zhang, Siyan Yi, Abhishek Shankar, Evlina Suzanna, So-Youn Jung, Peh Joo Ho, Aasim Yusuf, Ashrafun Nessa, Kyu-Won Jung, Eshani Fernando, Shweta Baral, Maryam Bagherian, Prabhat Pradhan, Uranbolor Jugder, Champadeng Vongdala, Siti Norbayah Yusof, Khin Thiri, Patumrat Sripan, Clarito Cairo, Tomohiro Matsuda, Suleeporn Sangrajran, Veronique Kiak-Mien Tan, Ravi Mehrotra, Benjamin O. Anderson

**Affiliations:** a PAPRSB Institute of Health Sciences, Universiti Brunei Darussalam, Brunei Darussalam; b Bureau of International Health Cooperation, National Center for Global Health and Medicine, Japan; c Faculty of Medicine, University of Malaya, Malaysia; d Center for Population Health Sciences, Hanoi University of Public Health, Hanoi, Vietnam; e Centre for Public Health, Queen's University Belfast, United Kingdom; f Women's Health and Genetics, Genome Institute of Singapore, A∗Star, Singapore; g School of Digital Science, Universiti Brunei Darussalam, Brunei Darussalam; h National Cancer Center/National Clinical Research Center for Cancer/Cancer Hospital, Chinese Academy of Medical Sciences & Peking Union Medical College, Beijing, China; i KHANA Center for Population Health Research, Phnom Penh, Cambodia; j Saw Swee Hock School of Public Health, National University of Singapore and National University Health System, Singapore, Singapore; k Department of Radiation Oncology, Dr BR Ambedkar Institute Rotary Cancer Hospital, All India Institute of Medical Sciences, Delhi, India; l National Cancer Center Indonesia, Dharmais Cancer Hospital, Jakarta, Indonesia; m Center for Breast Cancer, National Cancer Center, Goyang, Republic of Korea; n Shaukat Khanum Memorial Cancer Hospital and Research Centres, Lahore and Peshawar, Pakistan; o Department of Gynaecological Oncology, Bangabandhu Sheikh Mujib Medical University, Bangladesh; p National Cancer Centre Graduate School of Cancer Science and Policy, Goyang, Republic of Korea; q National Cancer Control Programme, Ministry of Health, Sri Lanka; r Bhaktapur Cancer Hospital, Bhaktapur, Nepal; s Breast Cancer Research Center, Motamed Cancer Institute, ACECR, Tehran, Iran; t Jigme Dorji Wangchuck National Referral Hospital, Bhutan; u Cancer Registry-surveillance and Early Detection Division, National Cancer Center of Mongolia, Ulaanbaatar, Mongolia; v Cancer Center, Mittaphab Hospital, Vientiane, Lao PDR; w National Cancer Institute, Putrajaya, Malaysia; x Pink Rose Breast Cancer Patients Support Group, Yangon, Myanmar; y Research Institute for Health Sciences, Chiang Mai University, Thailand; z Department of Health, Disease Prevention and Control Bureau, Manila, Philippines; aa National Cancer Center Institute for Cancer Control, Tokyo, Japan; ab National Cancer Institute, Bangkok, Thailand; ac National Cancer Centre Singapore, Singapore; ad Indian Cancer Genome Atlas, India & Centre for Health, Innovation and Policy Foundation, India; ae World Health Organisation (WHO), Geneva, Switzerland

**Keywords:** Breast cancer, WHO GBCI Framework, Feasibility, Indicators, Cancer stage, Survival, Screening, Early diagnosis, Completion of treatment, ANCCA, Asia

## Abstract

**Background:**

The Global Breast Cancer Initiative (GBCI) Framework, launched by the World Health Organisation (WHO) in 2023, emphasises assessing, strengthening, and scaling up services for the early detection and management of breast cancer. This study aims to determine the feasibility of monitoring the status of breast cancer control in the 21 Asian National Cancer Centers Alliance (ANCCA) countries based on the three GBCI Framework key performance indicators (KPIs): stage at diagnosis, time to diagnosis, and treatment completion.

**Methods:**

We reviewed published literature on breast cancer control among 21 ANCCA countries from May to July 2023 to establish data availability and compiled the latest descriptive statistics and sources of the indicators using a standardised data collection form. We performed bivariate Pearson's correlation analysis to measure the strength of correlation between stage at diagnosis, mortality and survival rates, and universal health coverage.

**Findings:**

Only 12 (57%) ANCCA member countries published national cancer registry reports on breast cancer age-standardised incidence rate (ASIR) and age-standardised mortality rate (ASMR). Indonesia, Myanmar, and Nepal had provincial data and others relied on WHO's Global Cancer Observatory (GLOBOCAN) estimates. GLOBOCAN data differed from the reported national statistics by 5–10% in Bhutan, Indonesia, Iran, the Republic of Korea, Singapore, and Thailand and >10% in China, India, Malaysia, Mongolia, and Sri Lanka. The proportion of patients diagnosed in stages I and II strongly correlated with the five-year survival rate and with the universal health coverage (UHC) index. Three countries (14%) reported national data with >60% of invasive breast cancer patients diagnosed at stages I and II, and a five-year survival rate of >80%. Over 60% of the ANCCA countries had no published national data on breast cancer staging, the time interval from presentation to diagnosis, and diagnosis to treatment. Five (24%) countries reported data on treatment completion. The definition of delayed diagnosis and treatment completion varied across countries.

**Interpretation:**

GBCI's Pillar 1 KPI correlates strongly with five-year survival rate and with the UHC index. Most ANCCA countries lacked national data on cancer staging, timely diagnosis, and treatment completion KPIs. While institutional-level data were available in some countries, they may not represent the nationwide status. Strengthening cancer surveillance is crucial for effective breast cancer control. The GBCI Framework indicators warrant more detailed definitions for standardised data collection. Surrogate indicators which are measurable and manageable in country-specific settings, could be considered for monitoring GBCI indicators. Ensuring UHC and addressing health inequalities are essential to early diagnosis and treatment of breast cancer.

**Funding:**

Funding for this research article's processing fee (APC) will be provided by the affiliated institution to support the open-access publication of this work. The funding body is not involved in the study design; collection, management, analysis and interpretation of data; or the decision to submit for publication. The funding body will be informed of any planned publications, and documentation provided.

**Research in context**

**Evidence before this study**

Cancer control programmes aim to reduce the disease’s burden and improve patients’ quality of life. It includes intervention programmes for prevention, early detection, diagnosis, treatment, and palliative care. The WHO’s Global Breast Cancer Initiative (GBCI) aims to reduce breast cancer mortality by 2.5% per year through three key strategies: health promotion for early detection, timely diagnosis, and comprehensive breast cancer management. Currently, no standardised system is available to collect and monitor the GBCI Framework Key Performance Indicators (KPIs).

**Added value of this study**

This study provides an overview of breast cancer burden and control measures in 21 Asian National Cancer Centres Alliance (ANCCA) countries, and the feasibility of monitoring the GBCI Framework KPIs in the countries. Key domains related to the feasibility of monitoring GBCI KPIs were identified. Only 12 (57%) ANCCA member countries published national cancer registry reports on breast cancer burden, most countries used WHO’s Global Cancer Observatory (GLOBOCAN) estimates. For countries with reported national statistics, GLOBOCAN estimates differed from the reported national statistics by 5-10% in Bhutan, Indonesia, Iran, Republic of Korea, Singapore, and Thailand and >10% in China, India, Malaysia, Mongolia, and Sri Lanka. The proportion of patients diagnosed in stage I and II strongly correlated with five-year survival rate and moderately with the universal health service coverage index. Data related to the time interval from first presentation to diagnosis and diagnosis to treatment were hardly available. The available data were mostly from the institutional level. A similar pattern was observed when examining the data related to the percentage of patients diagnosed within 60 days of presentation and those who successfully completed their treatment.

**Implications of all the available evidence**

Our findings inform the priority areas which policymakers should focus on, particularly in (i) ensuring universal health coverage, capacity building, and resources allocation for a timely diagnosis and comprehensive management; (ii) providing more detailed definitions for the GBCI Framework indicators for standardised data collection and (iii) developing breast cancer surveillance systems, which integrate indicators of the GBCI Framework in the routine data collection.

## Introduction

Globally, breast cancer is the most common cancer in women, with over 2.3 million new cases diagnosed every year. It ranks first or second in female cancer deaths in over 90% of countries.^1^ Over 70% of breast cancer deaths occur in low- and middle-income countries (LMICs) today.^1^ While high-income countries (HICs) have seen a 40% decline in breast cancer mortality over the past two decades, LMICs have not experienced a similar reduction.^2^ Breast cancer remains the leading cause of cancer-related deaths in women, especially in LMICs with underdeveloped healthcare systems, impacting families, communities, and economies significantly.^3^ Maternal cancer impacts about one million children worldwide, with breast cancer accounting for a quarter of the 4.4 million women who died from cancer in 2020.^4^ Children orphaned by maternal cancer face increased social, health, education, and financial risks.^4^ Breast cancer outcomes vary, with five-year survival rates ranging from over 90% in HICs to 40% in rural provinces in Asian LMICs.^5^

The World Health Organisation (WHO) launched the Global Breast Cancer Initiative (GBCI) Framework in 2023 to address these challenges. The framework aims to reduce breast cancer mortality by 2.5% annually and prevent 2.5 million breast cancer deaths globally by 2040. It focuses on early detection, timely diagnosis, and completion of breast cancer treatment as the fundamental pillars.^6^ Improving clinical outcomes and reducing mortality requires early detection and accessible diagnostic services and treatment options. The GBCI Framework establishes threshold targets for three key performance indicators (KPIs) corresponding to the three GBCI pillars: 1) diagnosing >60% of invasive breast cancers at stage I or II, 2) providing timely breast diagnostics within 60 days of initial presentation, and 3) ensuring that over 80% of patients undergo multimodality treatment without abandonment.^7^ The GBCI Framework emphasises assessing, strengthening, and scaling up services for the early detection and management of breast cancer.

By leveraging cost-effective, country-specific, and resource-appropriate measures, health promotion is integrated with primary healthcare services to ensure sustainability in achieving universal health coverage (UHC), especially in LMICs. KPIs are used to assess countries' progress in meeting these benchmarks. However, many Asian countries lack an adequate national surveillance system to monitor the cancer burden and evaluate breast cancer control programmes based on GBCI indicators. The Asian National Cancer Centers Alliance (ANCCA) was established in 2005 with members from Asian subregions. This study aims to assess the feasibility of monitoring the status of breast cancer control in the 21 ANCCA countries based on the GBCI Framework, to provide a snapshot of the status of breast control indicators, to discuss factors affecting breast cancer control and provide recommendations to improve breast cancer control among ANCCA members.

## Methods

### Country selections

This multi-country study was conducted from May to July 2023, focusing on 21 ANCCA member countries (Bangladesh, Bhutan, Brunei, Cambodia, China, India, Indonesia, Iran, Japan, Laos, Malaysia, Mongolia, Myanmar, Nepal, Pakistan, the Philippines, Singapore, the Republic of Korea, Sri Lanka, Thailand, and Vietnam). We performed a literature review to assess the feasibility of monitoring the GBCI Framework's KPIs in Asia, particularly among ANCCA countries. The study examined early detection, timely diagnosis, and multidisciplinary treatment. We examined published peer-reviewed and grey literature from individual countries to identify how these indicators are defined and measured at the country level.

### Search strategy

We identified published materials for the literature review by searching PubMed and Google Scholar using combinations of the search terms “breast cancer” ANDS “incidence” OR “mortality” OR “survival” OR “staging” OR “screening” OR “target age” OR “guideline” OR “delay” OR “diagnosis” OR “treatment” OR “barrier” OR “Asia” OR “Bangladesh” OR “Bhutan” OR “Brunei” OR “Cambodia” OR “China” OR “India” OR “Indonesia” OR “Iran” OR “Japan” OR “Korea” OR “Lao” OR “Malaysia” OR “Mongolia” OR “Myanmar” OR “Nepal” OR “Pakistan” OR “Philippines” OR “Singapore” OR “Sri Lanka” OR “Thailand” OR “Vietnam” from 2000 through 2023. We also searched authors' files, national and international health websites, and relevant national reports in their respective languages. The reference list was compiled based on the originality and relevance to the scope of this study.

### Data collection

A standardised data collection form (Supplementary Information) was used to compile the latest statistics and sources of indicators. We reviewed indicator definitions based on the GBCI Framework and international and national guidelines. The documents included staging systems (American Joint Committee on Cancer [AJCC] TNM^7^ or Surveillance, Epidemiology, and End Results [SEER]^8^) for breast cancer reporting, the time interval from presentation to diagnosis to treatment (median or mean), and the definition of treatment completion or compliance with clinical practice guidelines (CPGs) in the countries. Data sources were categorised as national, provincial, institutional, or facility levels. The form also included a short questionnaire identifying common and country-specific factors influencing breast cancer control. Data were provided by researchers comprised of epidemiologists, oncologists, and surgeons from each ANCCA country.

Age-standardised incidence and mortality rates for female breast cancers were collected from national or provincial cancer registries and the Global Cancer Observatory (GLOBOCAN) 2020 estimates. Information on breast cancer staging, five-year survival rates, awareness campaigns, screening guidelines, modalities and coverage, time intervals for diagnosis or treatment delays, and completion of multidisciplinary treatment were extracted from published literature, including reports from WHO and national government institutions, such as cancer registry reports on official websites. If national statistics were not available, we presented provincial or institutional statistics. In such cases, we prioritised studies from the latest period or with larger sample sizes.

### Statistical analyses

We performed bivariate Pearson's correlation analysis to measure the strength of the correlation between cancer staging, five-year survival rate, age-standardised mortality rate (ASMR), and UHC index.^9^ Data analysis was performed in R v.3.6.1. Pearson's correlations ranging from 0 to 0.19, 0.2 to 0.39, 0.40 to 0.59, 0.6 to 0.79 or 0.8 to 1 were classified as very weak, weak, moderate, strong and very strong, respectively.^10^ We ran the Shapiro–Wilk's normality test and inspected Q–Q plots using the *shapiro.test* and *qqPlot,* and confirmed normal distribution. We used the *wtd.cor* function with weights associated to the respective female population to check for added correlation. We also conducted sensitivity analyses to assess robustness of the findings with alternative assumptions on missing data (Supplementary Information Appendix D).

### Role of the funding source

The funding body is not involved in the study design; collection, management, analysis and interpretation of data; or the decision to submit for publication. The funding body will be informed of any planned publications, and documentation provided. Sokking Ong, Rei Haruyama and Cheng Har Yip have access to all the data collected for the study. All authors contributed to data interpretation and agreed to submit the final form of the manuscript.

## Results

### Malignant neoplasm of breast (ICD-10C50) burden among ANCCA members—GLOBOCAN estimates versus local registry data

**Fig. 1:**
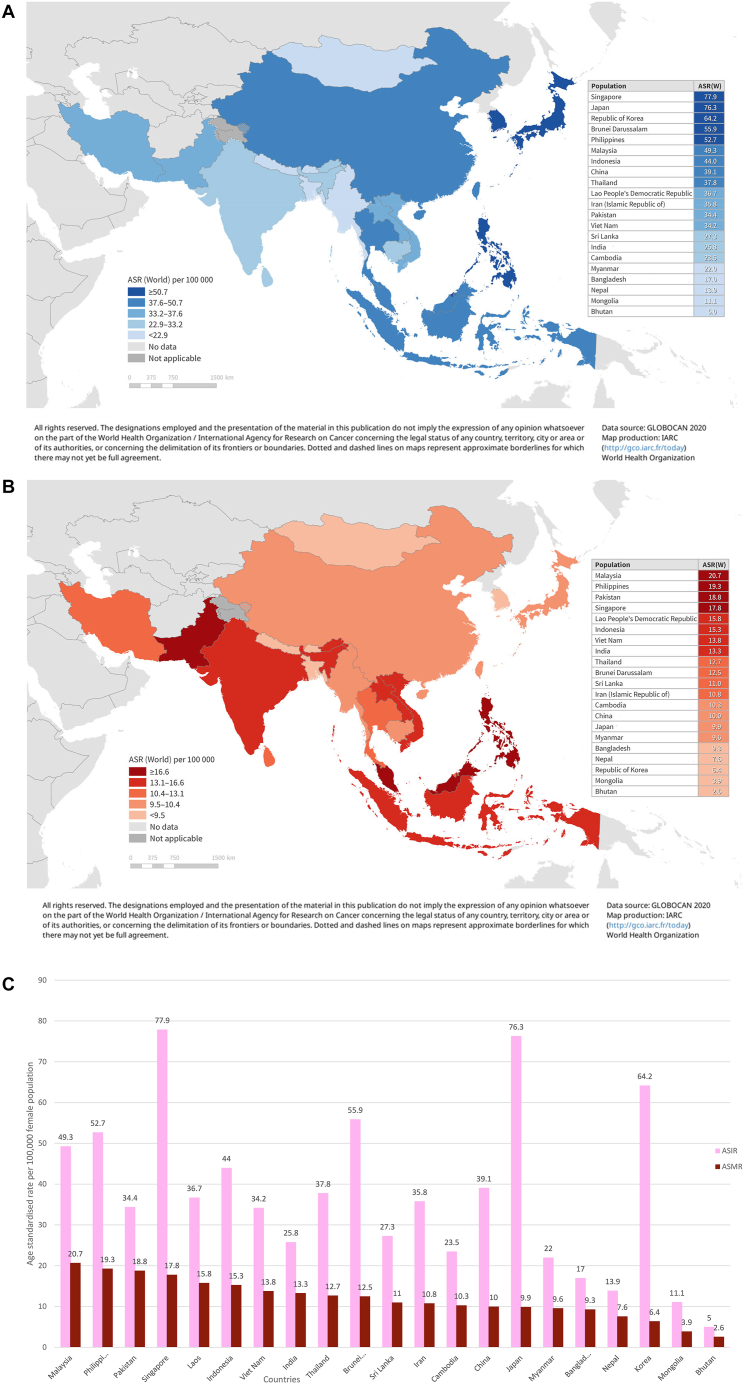
A) **Age-standardised incidence rate (ASIR) of breast cancer in 21 Asian National Cancer Centers Alliance (ANCCA) countries in a graphic map.** The graphic map highlighted the 21 Asian National Cancer Centers Alliance (ANCCA) countries in color according to their estimated age-standardised incidence rate (ASIR) of breast cancer per 100,000 female population. The lighter the blue color, the lower ASIR. The table attached to the map shows the 21 ANCCA countries in order from having the highest ASIR (Singapore) to having the lowest ASIR (Bhutan). B) **Age-standardised mortality rate (ASMR) of breast cancer in 21 Asian National Cancer Centers Alliance (ANCCA) countries in a graphic map**. The graphic map highlighted the 21 Asian National Cancer Centers Alliance (ANCCA) countries in color according to their estimated age-standardised mortality rate (ASMR) of breast cancer per 100,000 female population. The lighter the red color, the lower ASMR. The table attached to the map shows the 21 ANCCA countries in order from having the highest ASMR (Malaysia) to having the lowest ASMR (Bhutan). C) **Age-standardised incidence rate (ASIR) and Age-standardised mortality rate (ASMR) of breast cancer in 21 Asian National Cancer Centers Alliance (ANCCA) countries**. The bar chart compares the age-standardised incidence rate (ASIR) and age-standardised mortality rate (ASMR) of breast cancer per 100,000 female population across the 21 Asian National Cancer Centers Alliance (ANCCA) countries. The columns are arranged in descending order, from the country with the highest ASMR to the one with the lowest ASMR. Malaysia exhibits the highest ASMR, while Singapore leads in ASIR. The chart shows the regional variation of breast cancer incidence and mortality.

Of the 21 ANCCA countries, only 12 (57%) published national cancer registry reports on breast cancer age-standardised incidence rate (ASIR) and ASMR (Table 1). Three countries (Indonesia, Myanmar and Nepal) had provincial data, while others relied on GLOBOCAN estimates. GLOBOCAN data differed from the reported national statistics by 5–10% in six countries (Bhutan, Indonesia, Iran, the Republic of Korea, Singapore and Thailand) and >10% in five countries (China, India, Malaysia, Mongolia, and Sri Lanka). Significant heterogeneity in breast cancer burden was observed across Asia. ASIR ranged from five per 100,000 women in Bhutan to 78 per 100,000 women in Singapore, and ASMR ranged from three per 100,000 in Bhutan to 21 per 100,000 women in Malaysia (Fig. 1A, B and 1C). Large countries like China, India, and Indonesia showed substantial subnational variation, while Japan and the Republic of Korea had more consistent rates.^11^, ^12^, ^13^, ^14^, ^15^ In China, breast cancer was the most common female cancer in urban areas but the fourth in rural areas, with ASIR ranging from 47 cases per 100,000 women in Guangzhou to eight cases per 100,000 women in less developed regions.^16^

**Table 1:** Breast cancer incidence, mortality and 5-year survival indicators among ANCCA member countries.

	Population (thousand)	Female population (thousand)	GLOBOCAN 2020 estimates		National or provincial registry data			5-year survival	
			ASIR	ASMR	ASIR	ASMR	Data source	%	Data source
Bangladesh	166,427	85,358	17.0	9.3	NA	NA	NA	NA	NA
Bhutan	770	366	5.0	2.6	4.6	NA	National	61	Provincial
Brunei	440	215	55.9	12.5	57.9	14.0	National	72.0	National
Cambodia	16,296	8377	23.5	10.3	NA	NA	NA	NA	NA
China	1,423,998	697,843	39.1	10.0	29.1	6.4	National	82.0	National
India	1,389,966	681,060	25.8	13.3	30	NA	National	51	National
Indonesia	270,826	135,901	44.0	15.3	47.8	15.4	Provincial	77.7	Provincial
Iran	86,990	43,497	35.8	10.8	34.0	11.9	National	69.5	Provincial
Japan	125,543	64,045	76.3	9.9	77.1	8.6	National	92.3	National
Korea, Republic of	51,858	25,945	64.2	6.4	59.9	5.6	National	93.8	National
Lao PDR	7266	3682	36.7	15.8	NA	NA	NA	NA	NA
Malaysia	33,004	16,407	49.3	20.7	34.1	NA	National	66.8	National
Mongolia	3322	1686	11.1	3.9	16.1	5.8	National	76.1	National
Myanmar	53,228	27,015	22.0	9.6	22.4	17.9	Provincial	NA	NA
Nepal	28,999	15,664	13.9	7.6	13.7	7.2	Provincial	NA	NA
Pakistan	231,402	114,586	34.4	18.8	NA	NA	NA	NA	NA
Philippines	111,288	56,063	52.7	19.3	NA	NA	NA	59	National
Singapore	5894	2834	77.9	17.8	73.8	12.0	National	82.4	National
Sri Lanka	21,683	11,283	27.3	11.0	38.8	6.4	National	71.6 (localized)	National
Thailand	71,389	36,807	37.8	12.7	34.2	14.6	National	68.8	Provincial
Vietnam	96,204	49,332	34.2	13.8	NA	NA	NA	74	Provincial

ANCCA = Asian National Cancer Centers Alliance; ASIR = age-standardised incidence rate of breast cancer per 100,000 female population; ASMR = age-standardised mortality rate of breast cancer per 100,000 female population; NA = not available.

### Breast cancer awareness campaigns, screening guidelines and modalities, target age group, and uptake rates

Thirteen (62%) ANCCA countries regularly conducted national breast cancer awareness campaigns. Breast cancer screening and early diagnosis programmes were implemented in all ANCCA countries, but only eight (38%) had national-level programs, while the rest were at provincial or institutional levels. Breast cancer screening guidelines were available in 15 (71%) countries, with the target age group ranging from 20 to 70 years.

Six countries (Bhutan, Brunei, Japan, the Republic of Korea, Malaysia, and Singapore) used mammograms only, while nine countries (China, India, Indonesia, Iran, Laos, Nepal, the Philippines, Thailand, and Vietnam) offered mammograms along with ultrasound and/or clinical breast examination (CBE) (Table 2). Seven ANCCA countries reported a national screening uptake of more than 30% but below 70% (Table 2). The definition of screening uptake varied among countries, including having ever been screened, screened within a specific timeframe, and among eligible age groups.

**Table 2:** Breast cancer (BC) control indicators among ANCCA members.

	BC health campaign^a^	BC screening guideline	BC screening program implementation	Year screening program started	Target age group	Primary screening test offered	Screening uptake rate %	% of patients diagnosed in stage I and II	Median time interval from first presentation to diagnosis (days)	% of patients diagnosed ≤60–90 days of first presentation	Median time interval from diagnosis to treatment (days)	% of patients complete treatment	UHC service coverage index (0–100)
Bangladesh	N	Avail	N	2007	30–60	CBE	6.7	4^b^	NA	NA	NA	NA	51
Bhutan	O	Avail	P	2015	40–65	Mammo	NA	NA	NA	NA	NA	NA	62
Brunei	N	Avail	N	2019	40–69	Mammo	11	41	NA	NA	NA	NA	77
Cambodia	O	NA	P	2015	NA	NA	NA	22^b^	NA	NA	NA	NA	61
China	N	Avail	P	2012 (14 cities)	40–69	Mammo/US	31	73^b^	NA	60 (90 days)^b^	60^b^	NA	82
India	N	Avail	P	2010	30–65	CBE/Mammo	10^b^	29	30^b^	NA	130^b^	NA	61
Indonesia	P	NA	P	NA	≥40	CBE/US/Mammo	35	NA	30^b^	64 (60 days)^b^	NA	61.6^b^	59
Iran	N	Avail	N	2012	40–70	CBE/Mammo	16^b^	54^b^	NA	56 (60 days)^b^	NA	NA	77
Japan	N	Avail	N	2000	40+	Mammo	37	82	NA	NA	NA	NA	85
Korea, Republic of	N	Avail	N	2002	40–74	Mammo	64	72	NA	NA	14^b^	NA	87
Lao PDR	O	NA	O	NA	NA	CBE/Mammo	33^b^	67^b^	NA	NA	NA	NA	50
Malaysia	N	Avail	O	2002	50–74	Mammo	7–15^b^	52	26^b^	73 (90 days)^b^	21^b^	76^b^	76
Mongolia	N	Avail	N	2013	20–60	CBE	44	51	NA	NA	NA	NA	63
Myanmar	O	NA	O	NA	NA	CBE	NA	NA	NA	NA	NA	NA	61
Nepal	O	Avail	O	2010	20+	CBE/Mammo	3–14^b^	11^b^	NA	NA	2–62^b^	NA	53
Pakistan	O	NA	O	NA	NA	CBE	10^b^	42^b^	NA	NA	NA	NA	45
Philippines	N	NA	O	NA	NA	CBE/Mammo	NA	47^b^	NA	NA	NA	NA	55
Singapore	N	Avail	N	2002	50+	Mammo	38	77	7^b^, ^c^	95 (60 days)^b^	34^b^	64–90^b^	86
Sri Lanka	N	Avail	N	1996	35 and 45	CBE	37	66^b^	NA	NA	NA	59^b^	67
Thailand	N	Avail	P	2014	40–70	CBE/Mammo	2–29^b^	69^b^	NA	90 (60 days)^d^	NA	70	83
Vietnam	O	Avail	O	NA	40+	CBE/Mammo	25–51^b^	36^b^	72^b^	52 (90 days)^b^	93^b^	NA	70

ANCCA = Asian National Cancer Centers Alliance; Mammo = Mammogram; CBE = Clinical breast examination; US = Ultrasound; UHC = Universal Health Coverage.

a Program types: N = Nationwide; P = Provincial/Regional; O = Others e.g., ad-hoc, private, opportunistic. NA = Not available or no published data available.

b Provincial or institutional statistics from national cancer centers or hospitals.

c Median time measured from the first presentation at the main tertiary referral hospitals (e.g., surgical consultation) to definitive diagnosis.

d Proportion of patients diagnosed within 60 days measured from when pathology test was first ordered (at primary healthcare and tertiary institutions nationwide) to having a definitive diagnosis.

### Feasibility of monitoring GBCI Framework indicators

We identified four key domains in assessing the feasibility of monitoring GBCI Framework indicators among ANCCA countries, summarised as the four “As” in Table 3.

**Table 3:** Four key domains in assessing the feasibility of monitoring GBCI Framework indicators.

Domain	Description
Availability	Is the data available at the national, provincial, institutional, or facility level? Is the data validated, standardised, current, peer-reviewed, published, or unpublished?
Applicability	Is the available data utilised in evaluating breast cancer control in the setting? Is the available data integrated as part of the routine data collection of breast cancer control programmes in the country?
Adaptation	Is the available data adaptable to the GBCI Framework indicators? Is there a clear definition of the indicator or data collected?
Additional	Is additional information or processes required to collect national data? Is there any surrogate or proxy indicator e.g., the number of clinic visits needed for women to receive screening or diagnosis or treatment?

GBCI = Global Breast Cancer Initiative.

### Pillar 1 KPI target: >60% of invasive breast cancers are stage I or II at diagnosis

**Fig. 2:**
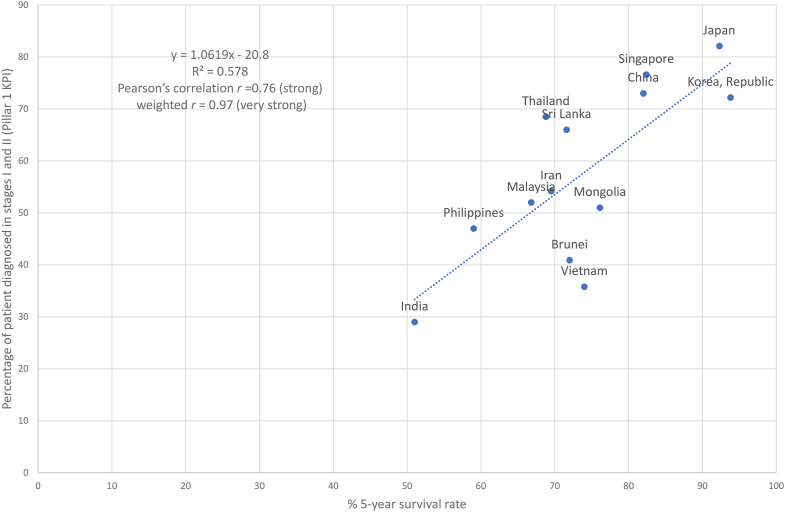
**Correlation between proportion of patients diagnosed at stages I and II (Pillar 1 KPI) and 5-year survival rate**. The dotted (regression) line represents the linear regression model (y = 1.0619x—20.8) that best fit the shown countries' proportion of patients diagnosed at stages I and II and 5-year survival rate, indicated by the blue dots. The graph shows that there is a positive linear relationship between proportion of patients diagnosed at stages I and II and 5-year survival rate as the value of one attribute increases, so does the other. The R^2^ value of 0.578 indicates a strong relationship.

**Fig. 3:**
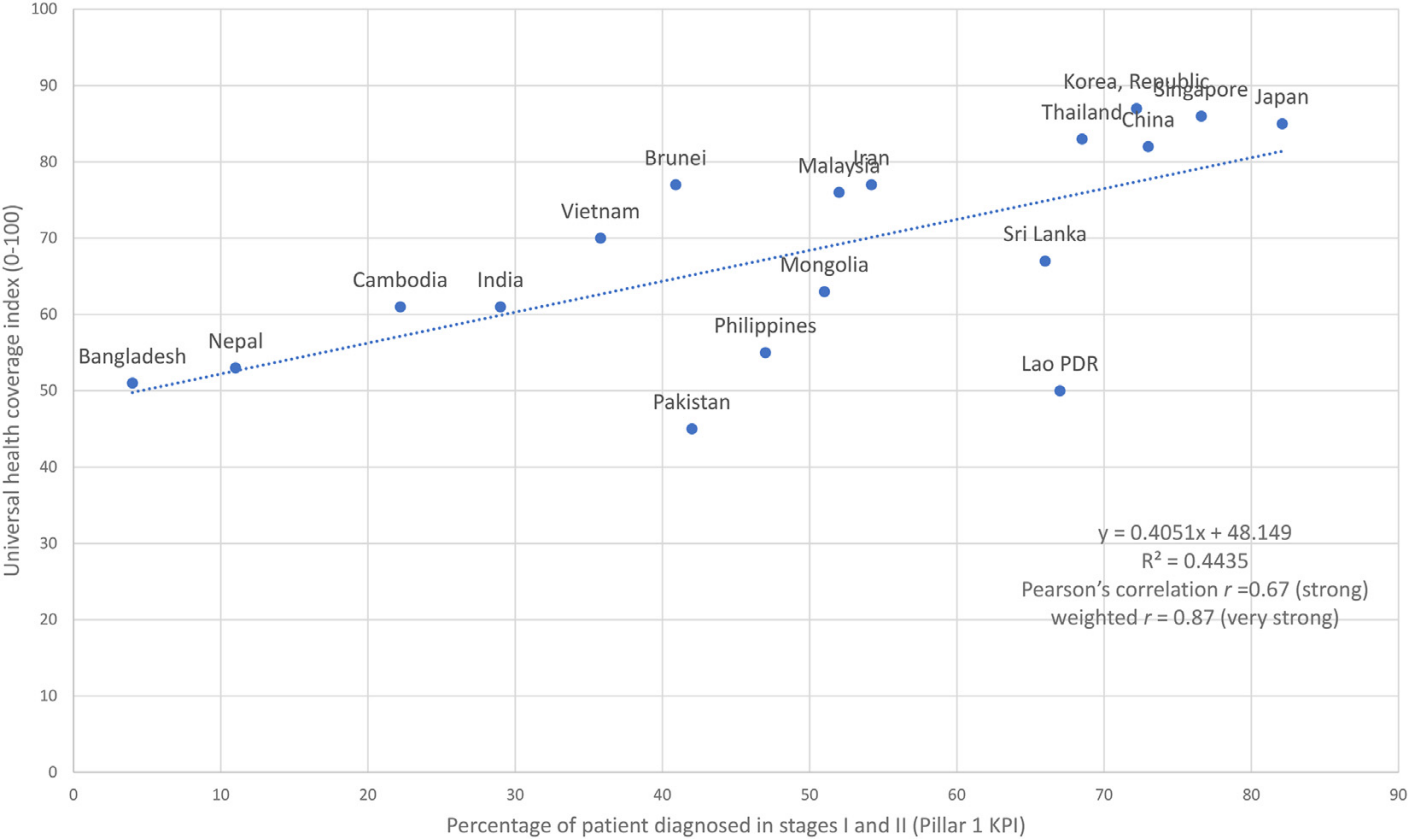
**Correlation between proportion of patients diagnosed at stages I and II (Pillar 1 KPI) and Universal Health Coverage index**. The dotted (regression) line represents the linear regression model (y = 0.4051x + 48.149) that best fit the shown countries' proportion of patients diagnosed at stages I and II (Pillar 1 KPI) and Universal Health Coverage index, indicated by the blue dots. The graph shows that there is a positive linear relationship between proportion of patients diagnosed at stages I and II (Pillar 1 KPI) and Universal Health Coverage index as the value of one attribute increases, so does the other. The R^2^ value of 0.4435 indicates a strong relationship.

Early detection can occur through mammographic and/or clinical screening (for eligible women without symptoms) or early diagnosis by clinical assessment (for women with suspected symptoms). Breast cancer stages I and II are defined by anatomic TNM staging of the AJCC.^17^ Some countries reported localised stages using the SEER staging system instead of AJCC TNM stages I and II. Seven (33%) ANCCA countries reported national cancer staging. Three ANCCA countries (Japan, the Republic of Korea, and Singapore) reported national data of having >60% of breast cancer patients diagnosed at the early stages (Table 2) and a five-year survival rate of >80% (Table 1). We found that Pillar 1 KPI correlates strongly with the five-year survival rate (r = 0.76, 95% CI [0.36, 0.92], Fig. 2), and the UHC index (r = 0.67, 95% CI [0.29, 0.86], Fig. 3), and weakly with ASMR (r ≤ 0.01, Appendix C Fig. 4a and b). For correlations weighted to respective female populations, the correlations strengthened for Pillar 1 KPI with five-year survival and UHC index (Figs. 2 and 3).

### Pillar 2 KPI target: diagnosing breast cancer within 60 days of initial presentation

The second pillar focuses on prompt diagnosis. Eight countries (38%) had reported data on the time interval from initial presentation to diagnosis or the percentage of patients diagnosed without delay. They were from institutional studies except for Thailand, where the data were obtained from the national reimbursement database. In Thailand, the dataset covered the timeframe from the initial request for a pathology test to the eventual confirmation of diagnosis. The definition of delayed diagnosis varied among studies, ranging from more than 30 to 90 days.^18^ Studies indicate that mortality significantly increases when the delay exceeds 60–90 days.^19^^,^^20^ Median time interval data from the first presentation to confirmed diagnosis were available in five countries. They were all from institutional studies. In the case of Singapore, the data measured the time interval from the initial consultation at a tertiary hospital to the biopsy date (i.e., time to presentation to surgeon and not primary care) (Table 2).

### Pillar 3 KPI target: >80% of patients complete their recommended treatment regimes

This indicator measures effective completion of treatment without abandonment. Treatments include surgery, radiotherapy, chemotherapy, targeted therapy, immunotherapy, hormone therapy, and supportive services. Prompt treatment without significant delay reflects the accessibility of the healthcare system. Seven countries (33%) reported a time interval from diagnosis to treatment and only five (24%) countries had reported data on treatment completion (Table 2). In HICs like Singapore, the delay between diagnosis and starting breast cancer treatment was minimal, with 85.4% of breast cancer patients receiving their first treatment in less than 60 days of diagnosis.^21^

### Common and country-specific factors affecting breast cancer control

Approximately two-thirds of ANCCA countries (n = 14, 67%) provided government subsidies to improve the affordability of breast cancer treatment. Eighteen countries (86%) identified shortages of healthcare facilities or trained professionals as barriers, especially in rural areas. Pre-treatment diagnosis using immunohistochemistry (e.g., estrogen receptor [ER], progesterone receptor [PR], human epidermal growth receptor-2 [HER2]) was common in 14 countries (67%), mainly in urban areas. Urban and rural healthcare disparities were noted in 14 countries (67%). Lack of awareness and knowledge, and psychological or cultural reasons were identified as barriers to breast cancer control in 19 countries (90%).

### Recommendations for monitoring GBCI Framework KPIs aimed at achieving breast cancer control

#### #1 Universal health coverage (UHC) for timely diagnosis and management

Ensuring UHC and addressing health inequalities are essential to early diagnosis and treatment of breast cancer. Breast health education to improve health literacy should be linked to women's health and reproductive services. Regular health campaigns in communities, schools, and workplaces raise awareness of breast health and cancer risk factors.^22^ Countries should prioritise resources in breast cancer early detection including but not limited to screening, consider sustainable financing to cover the cost of diagnosis and treatments, and implementing regulations to ensure timely access to health services to enhance early diagnosis and breast cancer management. Sustainable financing models for cancer care, such as Japan's social health insurance system^23^ and Singapore's compulsory government-administered medical savings scheme,^24^ play a crucial role in facilitating timely access to health facilities and reduce catastrophic out-of-pocket payments. A long-term, multi-pronged approach is recommended to address key factors and barriers in the healthcare system. Capacity-building efforts should encompass the availability of core biopsy for suspected breast lesions and receptor testing for women. Pillar 2 aims to have breast cancer evaluation, imaging, tissue sampling and pathology diagnosis within 60 days of the first presentation. It entails rapid diagnosis, potentially through ultrasound at secondary-level facilities and a patient navigation system to ensure a continuum of care from primary to secondary and tertiary care. The Philippines enacted the National Integrated Cancer Control Act in 2019, which mandates the creation and implementation of patient navigation program from the communities and primary care to tertiary health facilities.^25^ Supportive care, including antiemetic and psychological support, aids in completing the recommended treatment regimens.

#### #2 Definitions for GBCI Framework KPIs and other relevant indicators

The GBCI Framework KPIs need more detailed definitions for standardised data collection. The current definitions are general to allow for flexibility and variation among countries in their capacity for data measurement. ANCCA may develop and promote a consensus on more precise definitions for GBCI KPIs 2 and 3. This would allow the countries to monitor the indicators at major cancer care hospitals and benchmark the KPIs for the Asia regions. The 60-day benchmark for Pillar 2 KPI is based on a study showing lower survival rates with delays exceeding three months.^26^ Brazil mandates treatment waiting time not to exceed 60 days after diagnosis.^27^ Cancer centres should aim for a maximum 90-day delay from the first presentation to treatment.^18^^,^^19^ The initial presentation to the health system should be clearly defined as the first presentation at the primary healthcare settings. Pillar 3 KPI on completion of treatment requires a more detailed definition to include supportive and palliative care. Surrogate indicators which are measurable and manageable in country-specific settings can be considered for monitoring GBCI indicators. Furthermore, it is essential to monitor additional indicators on quality of care, such as addressing the axilla during breast surgery, to ensure the consistent delivery of high-quality care within the region.

#### #3 Cancer surveillance system

A surveillance system is vital, providing data to inform policymakers and measure breast cancer prevention and control progress. GLOBOCAN estimates may not be suitable for monitoring progress over time, countries without a population-based registry should consider establish one. In most HICs, the mandatory notification of cancer cases as notifiable diseases significantly enhances cancer surveillance and bolsters the comprehensiveness of cancer registry data. Staging information is essential in the evaluation of cancer downstaging and early detection programmes. Additionally, it would be informative to consider the proportion of cancer cases diagnosed at stage 0 or ductal carcinoma in-situ (DCIS) to evaluate the effectiveness of mammographic screening programmes. Even in countries with available cancer registries, screening registries or records are often not interlinked, so the exact participation rate remains unknown to the municipalities or the central level. There is variation in programme management among municipalities (e.g., an invitation for screening, recall, compliance with the national standard, and tracking of the screen positives). Therefore, the ministries of health or the national health systems play a crucial role in gathering and analysing data from different institutions, using unique identifications and computerised records at hospitals and government offices. The WHO International Agency for Research on Cancer offers guidelines and training modules to develop cancer registration systems according to different levels of resource settings. Multi-country observational studies using a standardized protocol may be conducted to understand the baseline data of these indicators at 1–2 main cancer centres of each country.

Our recommendations are interconnected. UHC ensures access to healthcare services including cancer care and surveillance. It also provides a clear monitoring framework to guide policy development and evaluation of the healthcare systems. Their impact synergises with their shared goal of reducing the breast cancer burden and improving patients’ outcomes. Implementing these recommendations especially in resource-constrained settings, requires situation analysis and consideration of local contexts. It may require adjusted approaches, such as tailoring a phased implementation, leveraging technology for cost-effective solutions, cross-sectoral collaboration, training and capacity building for primary healthcare, pooling of resources, and advocacy for funding.

## Discussion

We assessed the feasibility of monitoring breast cancer control in the 21 ANCCA countries based on the GBCI Framework and provided an overview of the current reporting of breast cancer KPIs to determine if countries have achieved the WHO GBCI Framework targets. Our findings indicated significant challenges in monitoring breast cancer control activities, with over 60% of the ANCCA countries lacking national data on cancer staging and the time interval from presentation to diagnosis or treatment. While institutional-level data were available in some countries, they may not represent the nationwide status.

Population-based cancer registries are essential for understanding disease burden, setting national priorities, and monitoring cancer control efforts.^28^ Encouragingly, most ANCCA countries had national or provincial cancer registry data on breast cancer incidence. However, cancer reporting is not mandated in many countries, leading to the potential underreporting of cases, particularly in rural areas or from private hospitals. The accuracy of mortality statistics may also be affected by inaccurate recording of causes of death by laymen (e.g., police officers).^29^^,^^30^ The underreported cancer cases and lack of accurate mortality statistics have significant implications for interpreting ASIR and ASMR. The actual magnitude of the breast cancer burden may be underestimated, making it difficult to accurately assess the effectiveness of breast cancer control efforts. Environmental risk factors, such as diet in the population, also influence ASIR and ASMR. Therefore, understanding and addressing these risk factors are critical components of breast cancer control strategies in the region. As breast cancer incidence and mortality rates remain high in Asia, the potential for significant reduction is likely to be seen in areas with higher ASMR. The high rates reflect delays in presentation, diagnosis, and completion of treatment, indicating the areas where breast cancer control efforts can have the most impact.

GLOBOCAN data are valuable for estimating the cancer burden in a country. However, in countries where local data are unavailable, GLOBOCAN estimates are calculated using data from neighbouring countries with similar socioeconomic or health profiles. We observed differences between GLOBOCAN estimates and country-reported ASIR and ASMR (Table 1). GLOBOCAN's modelling multiplies the latest cancer incidence and mortality rates by the future population, assuming that the current incidence and mortality risk will remain constant. Therefore, this approach may not capture the latest information on cancer burden from the individual countries.

The GBCI Framework Pillar 1 KPI focuses on the proportion of invasive breast cancer cases diagnosed at stages I and II. Studies have shown that countries with sustained mortality reduction had at least 60% of breast cancer diagnosed in stage I or II.^31^ While mammography offers higher sensitivity for detecting breast cancer than does CBE, its usage for population-based screening is impractical in settings with limited resources.^32^ In such settings where late-stage breast cancer diagnoses are common, CBE performed by trained healthcare professionals is a fiscally realistic approach for identifying symptomatic cancers and improving early breast cancer diagnosis rates.^33^

Most ANCCA countries do not have published data on Pillars 2 and 3 KPIs, which are particularly relevant at the institutional level as part of quality improvement programming to determine if excessive delays occur or if a high fraction of patients are not completing their recommended therapies. Collectively, institutional data can be gathered to determine if obstacles to care are prevalent at the national level. These indicators regarding prompt diagnosis may not be routinely collected or reported, even at the institutional level, which could result in challenges in effectively planning and monitoring health system improvement programmes.

Additionally, it is essential to consider the extent of under-coverage for those not represented in the statistics or reporting system. Differences between urban and rural areas are expected in large countries like China, India, and Indonesia. Lack of screening facilities and accessibility are key barriers for women in rural areas to get screened. In rural areas, poor health literacy, belief in alternative therapy, cancer fatalism, and lack of autonomous decision-making have been reported in many Asian countries.^34^ Even in HICs like Singapore, where multiple channels for screening are available, screening uptake remains low indicating psychosocial barriers in these settings,^35^ fear of the financial burden associated with the costs of breast cancer treatment has been reported, as the government adopts a co-payment mode for health services.^36^^,^^37^

Our findings indicate that a higher proportion of women diagnosed at an early stage corresponds to better survival and lower mortality rates, consistent with other studies.^38^^,^^39^ However, in many LMICs, women are diagnosed at advanced stages due to limited access to facilities and sociocultural factors. The lack of information on the number of women not seeking diagnosis makes it hard to assess the full magnitude. Therefore, when countries implement screening programmes, an increase in the number of cases, including those in advanced stages, may be observed in the early phase. Favourable stage-shifting may only be achieved when early detection programmes have achieved high coverage (at least 60–70% of the target group). Targeted approaches for vulnerable or marginalised groups, including those relying on traditional medicines, are crucial. Culturally appropriate interventions should consider ethnic differences and empower women in these communities. Providing adequate education on the early detection of breast cancer to primary healthcare providers, such as the WHO package of essential noncommunicable disease interventions for primary health care (PEN) breast education module for primary healthcare^40^ and CBE training for district health providers, is essential, in addition to retaining skilled personnel in the country.

Pillar 3 KPI on the completeness of treatment warrants further clarification and specification at the country level. Completion of treatment can be measured as adherence or compliance with local CPGs, which may vary across countries and institutions. Variation in treatment recommendations across countries poses challenges to establishing a universal definition. For example, anti-HER2 therapies for HER2-overexpression breast cancers are recommended in HICs' CPGs but are not included in most LMICs due to high costs. In Malaysia, only 19% of HER2+ breast cancer patients received Herceptin, despite its CPG recommendation.^41^ In addition, the degree to which patients discontinue or abandon treatment without receiving the entire therapeutic course may go unrecognized, as is the case in multiple sub-Saharan African countries.^42^

Treatment completion without abandonment is affected by treatment affordability, supportive care availability, and side effect tolerance. The use of supportive care medications can reduce treatment interruptions. For example, granulocyte colony-stimulating factor (G-CSF) given prophylactically to prevent neutropenia associated with chemotherapy is more common in high-resourced settings. In some lower resource settings, antiemetics are not routinely provided to manage nausea and vomiting, which could lead to treatment discontinuation. Patients with ER-positive breast cancer are advised to undergo hormonal therapy for 5–10 years (depending on the cancer stage) but many discontinue prematurely due to side effects.^43^ Genetic variations in pharmacogenetics can impact Tamoxifen tolerance.^44^ This should be included in the treatment completeness definitions, especially if a change in therapy occurs due to severe side effects. In settings where a significant proportion of cancer is diagnosed in advanced stages, in addition to addressing breast cancer early diagnosis services, providing supportive or palliative care is crucial to reduce physical and psychological suffering. Effective treatment requires coordination among healthcare professionals for better outcomes. Private or non-governmental organisations' involvement can cause discrepancies between institutional data and population situations. Countries without published data on treatment completion rates could consider adopting the WHO's UHC index as a proxy indicator, as it is significantly associated with lower breast cancer mortality rates.^24^ Our study found strong correlations for GBCI Pillar 1 KPI with five-year survival and UHC index, the correlation strengthened with weightage to respective female populations.

In Asia, financial and social factors were commonly reported barriers to cancer screening, early detection, and treatment.^45^ The lack of accessibility and facilities for diagnosis and treatment is prevalent in rural and low-resource settings. About two-thirds of ANCCA member countries reported government subsidies for breast cancer treatment. However, even in countries with subsidies, a significant proportion of healthcare expenses is out-of-pocket or covered by private insurance. Cancer treatment often leads to significant financial hardships for the patients and families, with nearly half the patients in LMICs reporting an inability to pay for medicine in an ASEAN (ACTION) study.^46^

We identified a number of key strengths and limitations in our study. This study provides the first multi-country review of data availability on GBCI KPIs and breast cancer control in 21 Asian countries. ANCCA has the potential to assume a pivotal role in facilitating consensus-building and providing scientific recommendations for national cancer control initiatives. We explored monitoring feasibility and identified factors affecting breast cancer control. However, we should also consider the data quality and consistency of the country estimates–a low or high ASMR could result from underreporting or misclassification. Data quality inconsistencies are often more pronounced at the local or provincial levels, particularly in resource-constrained settings. Further studies are needed to investigate the sustainability and consistency of data collection, and if the setting is more conducive for continuous or episodic data collection, which could be linked to evaluating interventions for breast cancer control. This study presented the five-year survival rates. However, survival rates may extend beyond the five-year reporting, and there is an increasing trend in reporting 10- or 20-year survival rates, reflective of advancement in breast cancer treatment. In addition, no target indicators were identified for screening uptake to reduce breast cancer mortality, whereas a global target of 70% screening uptake was set to attain cervical cancer elimination.^47^ Our study is also limited by the inconsistency of data collection in the last few years due to the COVID-19 pandemic, which has impacted the implementation of breast cancer awareness campaigns, surveillance, screening, diagnosis, and treatment programmes.^48^

In conclusion, most ANCCA countries lacked national data on cancer staging (Pillar 1), timely diagnosis (Pillar 2), and treatment completion (Pillar 3) KPIs. Nearly half of the ANCCA countries GLOBOCAN estimates. For countries with reported national cancer statistics, GLOBOCAN estimates differed from the reported national statistics by 5–10% in Bhutan, Indonesia, Iran, Republic of Korea, Singapore, and Thailand and >10% in China, India, Malaysia, Mongolia and Sri Lanka. Data on Pillars 1 and 2 KPIs were mostly institutional. While institutional-level data were available in some countries, they may not represent the nationwide status. Strengthening cancer surveillance is crucial for effective breast cancer control. The GBCI Framework indicators warrant more detailed definitions for standardised data collection. Surrogate indicators which are measurable and manageable in country-specific settings, could be considered for monitoring GBCI indicators.

## Contributors

Conceptualization: Sokking Ong, Cheng Har Yip, Benjamin O. Anderson. Original draft: Sokking Ong. Data curation: Sok King Ong, Rei Haruyama, Cheng Har Yip, Yawei Zhang, Jingmei Li, Tran Thu Ngan, Peh Joo Ho, Evlina Suzanna, Kyu-Won Jung, Suleeporn Sangrajran, Ashrafun Nessa, Eshani Fernando, So-Youn Jung, Uranbolor Jugder, Abhishek Shankar, Maryam Bagherian, Aasim Yusuf, Prabhat Pradhan, Shweta Baral, Clarito Cairo, Khin Thiri, Champadeng Vongdala, Patumrat Sripan, Veronique Tan Kiak Mien, Ravi Mehrotra, Tomohiro Matsuda, Daphne Lai, Siyan Yi. Review, edits & revision: Sok King Ong, Rei Haruyama, Cheng Har Yip, Yawei Zhang, Jingmei Li, Tran Thu Ngan, Siyan Yi, Daphne Lai, Peh Joo Ho, Siti Norbayah Yusof, Abhishek Shankar, Tomohiro Matsuda, Veronique Tan Kiak Mien, Suleeporn Sangrajran, Ravi Mehrotra, Benjamin O. Anderson. Sokking Ong, Rei Haruyama and Cheng Har Yip have access to all the data collected for the study. All authors contributed to data interpretation and agreed to submit the final form of the manuscript.

## Data sharing statement

This study follows the Guidelines for Accurate and Transparent Health Estimates Reporting (GATHER). The findings of our study are supported by data available in public online repositories, data publicly available upon request of the data provider, and data not publicly available due to restrictions by the data provider. Non-publicly available data were used for the current study but may be available from the authors upon reasonable request and with permission of the data provider.

## Declaration of interests

The authors have no conflicts of interest to declare. The authors alone are responsible for the views expressed in this publication and they do not necessarily represent the decisions or policies of their affiliated institutions. Where authors are identified as personnel of the World Health Organisation (WHO), the authors alone are responsible for the views expressed in this article and they do not necessarily represent the decisions, policy or views of the WHO. Where maps are concerned, all rights are reserved by the WHO. The designations employed and the presentation of the material in this publication do not imply the expression of any opinion whatsoever on the part of the WHO concerning the legal status of any country, territory, city or area or of its authorities, or concerning the delimitation of its frontiers or boundaries. Dotted and dashed lines on maps represent approximate borderlines for which there may not yet be full agreement.

## Appendix A Supplementary data

Supplementary Appendix B

Supplementary Appendix C Fig 4: a) The dotted (regression) line represents the linear regression model (y = 0.0312x + 59.262) that best fit the shown countries’ proportion of patients diagnosed in stages I and II (Pillar 1 KPI) and local registry Age Standardised Mortality Rate (ASMR) of breast cancer per 100,000 female population**,** indicated by the blue dots. The graph shows that there is a positive linear relationship between proportion of patients diagnosed in Stage I and II (Pillar 1 KPI) and local registry’s ASMR as the value of one attribute increases, the other remain almost constant. The R^2^ value of 3E-05 indicates a very weak relationship. b) The dotted (regression) line represents the linear regression model (y = 0.535x + 43.04) that best fit the shown countries’ proportion of patients diagnosed in Stage I and II (Pillar 1 KPI) and GLOBOCAN 2020 estimates on Age Standardised Mortality Rate (ASMR) per 100,000 female population, indicated by the blue dots. The graph shows that there is a positive linear relationship between proportion of patients diagnosed in Stage I and II (Pillar 1 KPI) and GLOBOCAN 2020 estimates of ASMR as the value of one attribute increases, so does the other. The R^2^ value of 0.0117 indicates a very weak relationship. Fig 5: a) The dotted (regression) line represents the linear regression model (y = -1.0712x + 89.407) that best fit the shown countries’ 5-year survival rate and local registry age standardised mortality rate ASMR) per 100,000 female population**,** indicated by the blue dots. The graph shows that there is a negative linear relationship between 5-year survival rate and local registry’s ASMR per 100,000 female population as the value of one attribute increases, the other decreases. The R^2^ value of 0.2202 indicates a very moderate relationship. b) The dotted (regression) line represents the linear regression model (y = -0.3294x + 76.786) that best fit the shown countries’ Universal Health Coverage index and the age standardised mortality rate (ASMR) per 100,000 female population from the national or local cancer registry**,** indicated by the blue dots. The graph shows that there is a negative linear relationship between Universal Health Coverage index and local registry’s ASMR per 100,000 female population as the value of one attribute increases, the other decreases. The R^2^ value of 0.014 indicates a very weak relationship. c) The dotted (regression) line represents the linear regression model (y = -0.2308x + 69.954) that best fit the shown countries’ UHC service coverage index and GLOBOCAN 2020 estimates of Age Standardised Mortality Rate (ASMR) per 100,000 female population**,** indicated by the blue dots. The graph shows that there is a linear relationship between UHC service coverage index and GLOBOCAN 2020 estimates of ASMR as the value of one attribute increases, the other decreases. The R^2^ value of 0.0073 indicates a very weak relationship. d) The dotted (regression) line represents the linear regression model (y = -0.5277x + 79.532) that best fit the shown countries’ 5-year survival rate and GLOBOCAN 2020 estimates of Age Standardised Mortality Rate (ASMR) per 100,000 female population**,** indicated by the blue dots. The graph shows that there is a negative linear relationship between 5-year survival rate and the GLOBOCAN 2020 estimates of ASMR as the value of one attribute increases, the other decreases. The R^2^ value of 0.055 indicates a weak relationship.

## References

[1] World Health Organization International Agency for Research on Cancer (IARC) (2020). Globocan 2020: estimated cancer incidence, mortality and prevalence worldwide in 2020.

[2] Arnold M., Morgan E., Rumgay H. (2022). Current and future burden of breast cancer: global statistics for 2020 and 2040. Breast.

[3] Lim Y.X., Lim Z.L., Ho P.J., Li J. (2022). Breast cancer in Asia: incidence, mortality, early detection, mammography programs, and risk-based screening initiatives. Cancers.

[4] Guida F., Kidman R., Ferlay J. (2022). Global and regional estimates of orphans attributed to maternal cancer mortality in 2020. Nat Med.

[5] Allemani C., Matsuda T., Di Carlo V. (2018). Global surveillance of trends in cancer survival 2000-14 (CONCORD-3): analysis of individual records for 37 513 025 patients diagnosed with one of 18 cancers from 322 population-based registries in 71 countries. Lancet.

[6] (2023). Global Breast Cancer Initiative Implementation Framework: assessing, strengthening and scaling-up of services for the early detection and management of breast cancer.

[7] Amin M.B., Greene F.L., Edge S.B. (2017). The Eighth Edition AJCC Cancer Staging Manual: continuing to build a bridge from a population-based to a more "personalized" approach to cancer staging. CA Cancer J Clin.

[8] NIH NCI Surveillance Epidemiology, and End results program. Breast: summary stage manual 2018 v3.0. https://seer.cancer.gov/tools/ssm/SSM2018-BREAST.pdf.

[9] Tracking Universal Health Coverage (2021).

[10] T D V Swinscow (1997).

[11] Mehrotra R., Yadav K. (2022). Breast cancer in India: present scenario and the challenges ahead. World J Clin Oncol.

[12] Lei S., Zheng R., Zhang S. (2021). Breast cancer incidence and mortality in women in China: temporal trends and projections to 2030. Cancer Biol Med.

[13] Ng B., Puspitaningtyas H., Wiranata J.A. (2023). Breast cancer incidence in Yogyakarta, Indonesia from 2008-2019: a cross-sectional study using trend analysis and geographical information system. PLoS One.

[14] Cancer Statistics (2019). 2016-2019. Cancer information service, National Cancer Center, Japan [National Cancer Registry, Ministry of Health, Labour and Welfare].

[15] National Cancer Center (2022). https://ncc.re.kr/cancerStatsView.ncc?bbsnum=618&searchKey=total&searchValue=&pageNum=1.

[16] Fan L., Strasser-Weippl K., Li J.J. (2014). Breast cancer in China. Lancet Oncol.

[17] Hortobagyi G.N., Connolly J.L., D'Orsi C.J., Amin M.B., Edge S.B., Greene F.L. (2017). AJCC cancer staging manual.

[18] Sobri F.B., Bachtiar A., Panigoro S.S. (2021). Factors affecting delayed presentation and diagnosis of breast cancer in Asian developing countries women: a systematic review. Asian Pac J Cancer Prev.

[19] Trapani D., Ginsburg O., Fadelu T. (2022). Global challenges and policy solutions in breast cancer control. Cancer Treat Rev.

[20] Shih N.C., Kung P.T., Kuo W.Y., Tsai W.C. (2022). Association of treatment delay and stage with mortality in breast cancer: a nationwide cohort study in Taiwan. Sci Rep.

[21] Ho P.J., Cook A.R., Binte Mohamed Ri N.K., Liu J., Li J., Hartman M. (2020). Impact of delayed treatment in women diagnosed with breast cancer: a population-based study. Cancer Med.

[22] Akhtari-Zavare M., Juni M.H., Said S.M., Ismail I.Z., Latiff L.A., Ataollahi Eshkoor S. (2016). Result of randomized control trial to increase breast health awareness among young females in Malaysia. BMC Publ Health.

[23] Ministry of Health Labour and welfare. Health and medical services, Japan. http://www.mhlw.go.jp/english/policy/health-medical/health-insurance/index.html.

[24] Ministry of Health Singapore Healthcare schemes and subsidies. https://www.moh.gov.sg/healthcare-schemes-subsidies.

[25] Congress of the Philippines Metro Manila. The 2018 Philippines cancer bill. https://www.officialgazette.gov.ph/downloads/2019/02feb/20190214-RA-11215-RRD.pdf.

[26] Richards M.A., Westcombe A.M., Love S.B., Littlejohns P., Ramirez A.J. (1999). Influence of delay on survival in patients with breast cancer: a systematic review. Lancet.

[27] Ferreira N.A.S., Schoueri J.H.M., Sorpreso I.C.E., Adami F., Dos Santos Figueiredo F.W. (2020). Waiting time between breast cancer diagnosis and treatment in Brazilian women: an analysis of cases from 1998 to 2012. Int J Environ Res Public Health.

[28] World Health Organization (2020). https://apps.who.int/iris/handle/10665/330745.

[29] Wahab A., Choiriyyah I., Wilopo S.A. (2017). Determining the cause of death: mortality surveillance using verbal autopsy in Indonesia. Am J Trop Med Hyg.

[30] GBD 2016 Causes of Death Collaborators (2017). Global, regional, and national age-sex specific mortality for 264 causes of death, 1980-2016: a systematic analysis for the Global Burden of Disease Study 2016. Lancet.

[31] Duggan C., Trapani D., Ilbawi A.M. (2021). National health system characteristics, breast cancer stage at diagnosis, and breast cancer mortality: a population-based analysis. Lancet Oncol.

[32] Lauby-Secretan B., Scoccianti C., Loomis D. (2015). Breast-cancer screening--viewpoint of the IARC working group. N Engl J Med.

[33] World Health Organization (2014). http://www.who.int/cancer/publications/mammography_screening/en.

[34] Taib N.A., Yip C.H., Low W.Y. (2013). A grounded explanation of why women present with advanced breast cancer. World J Surg.

[35] Rajendram P., Singh P., Han K.T. (2022). Barriers to breast cancer screening in Singapore: A literature review. Ann Acad Med Singap.

[36] Lim S.K., Teo X.L., Ng J.L., Li F.X., Tan S.M. (2015). A survey on Singaporean women's knowledge, perception and practices of mammogram screening. Ann Acad Med Singap.

[37] Ng C.W.Q., Lim J.N.W., Liu J., Hartman M. (2020). Presentation of breast cancer, help seeking behaviour and experience of patients in their cancer journey in Singapore: a qualitative study. BMC Cancer.

[38] Mittra I., Mishra G.A., Dikshit R.P. (2021). Effect of screening by clinical breast examination on breast cancer incidence and mortality after 20 years: prospective, cluster randomised controlled trial in Mumbai. BMJ.

[39] Taylor C., McGale P., Probert J. (2023). Breast cancer mortality in 500 000 women with early invasive breast cancer in England, 1993-2015: population based observational cohort study. BMJ.

[40] World Health Organization (2013).

[41] Lim G.C., Aina E.N., Cheah S.K. (2014). Closing the global cancer divide--performance of breast cancer care services in a middle income developing country. BMC Cancer.

[42] Foerster M., McCormack V., Anderson B.O. (2022). Treatment guideline concordance, initiation, and abandonment in patients with non-metastatic breast cancer from the African Breast Cancer-Disparities in Outcomes (ABC-DO) cohort in sub-Saharan Africa: a prospective cohort study. Lancet Oncol.

[43] Rosso R., D'Alonzo M., Bounous V.E. (2023). Adherence to adjuvant endocrine therapy in breast cancer patients. Curr Oncol.

[44] Schroth W., Goetz M.P., Hamann U. (2009). Association between CYP2D6 polymorphisms and outcomes among women with early stage breast cancer treated with tamoxifen. JAMA.

[45] Mohan D., Su T.T., Donnelly M. (2021). Breast cancer screening in semi-rural Malaysia: utilisation and barriers. Int J Environ Res Public Health.

[46] ACTION Study Group (2017). Policy and priorities for national cancer control planning in low- and middle-income countries: lessons from the Association of Southeast Asian Nations (ASEAN) Costs in Oncology prospective cohort study. Eur J Cancer.

[47] Ong S.K., Abe S.K., Thilagaratnam S. (2023). Towards elimination of cervical cancer–human papillomavirus (HPV) vaccination and cervical cancer screening in Asian National Cancer Centers Alliance (ANCCA) member countries. Lancet Reg Health West Pac.

[48] Figueroa J.D., Gray E., Breast Screening Working Group (WG2) of the Covid-19 and Cancer Global Modelling Consortium (2021). The impact of the Covid-19 pandemic on breast cancer early detection and screening. Prev Med.

