# Supplementary material for: Feasibility of monitoring Global Breast Cancer Initiative Framework key performance indicators in 21 Asian National Cancer Centers Alliance member countries
Source: eClinicalMedicine. 2023 Dec 16;67:102365. doi: 10.1016/j.eclinm.2023.102365 (PMC10731600; doi:10.1016/j.eclinm.2023.102365)
Supplement: Supplementary Appendix A [file mmc1.docx]

**Appendix A: References list for Tables 1 and 2**

**For data in Table 1:**

1. United Nations Department of Economic and Social Affairs, Population Division (2022). Compact Demographic Indicators. <https://population.un.org/wpp/Download/Standard/MostUsed/> [Accessed 19 March 2023]
2. United Nations, Department of Economic and Social Affairs, Population Division (2022). World Population Prospects 2022, Online Edition. [Accessed 10 June 2023]
3. GLOBOCAN 2020. World Health Organization International Agency for Research on Cancer (IARC). GLOBOCAN 2020: estimated cancer incidence, mortality and prevalence worldwide in 2020.

Ferlay J, Ervik M, Lam F, Colombet M, Mery L, Piñeros M, Znaor A, Soerjomataram I, Bray F (2020). Global Cancer Observatory: Cancer Today. Lyon, France: International Agency for Research on Cancer. Available from: https://gco.iarc.fr/today, accessed [08 November 2023].

Sung H, Ferlay J, Siegel RL, Laversanne M, Soerjomataram I, Jemal A, Bray F. Global cancer statistics 2020: GLOBOCAN estimates of incidence and mortality worldwide for 36 cancers in 185 countries. CA Cancer J Clin. 2021 Feb 4. doi: 10.3322/caac.21660. Epub ahead of print. PMID: 33538338.

Ferlay J, Colombet M, Soerjomataram I, Parkin DM, Piñeros M, Znaor A, Bray F. Cancer statistics for the year 2020: An overview. Int J Cancer. 2021 Apr 5. doi: 10.1002/ijc.33588. Epub ahead of print. PMID: 33818764.

Ministry of Health Bhutan. Guideline For Screening Gastric Cancer, Cervical Cancer & Breast Cancer Available at [https://www.moh.gov.bt/wp-content/uploads/ict-files/2021/06/GUIDELINE-FOR-SCREENING-GASTRIC-CERVICAL-AND-BRTEAST-CANCERS.pdf Accessed 23 March 2023](https://www.moh.gov.bt/wp-content/uploads/ict-files/2021/06/GUIDELINE-FOR-SCREENING-GASTRIC-CERVICAL-AND-BRTEAST-CANCERS.pdf%20Accessed%2023%20March%202023)

Cancer Registry 2014 to 2022. Jigme Dorji Wangchuck National Referral Hospital, Thimphu, Bhutan. (unpublished data)

Leong E, Ong SK, Si‑Ramlee KA, Naing L. Cancer incidence and mortality in Brunei Darussalam, 2011 to 2020. BMC Cancer (2023) 23:466 <https://doi.org/10.1186/s12885-023-10962-8>

1. Leong E, Madli F, Ong SK. (2019). Five-year survival rate of breast cancer patients in Brunei Darussalam. Brunei International Medical Journal. 15. 73-81.

Zheng R, Zhang S, Zeng H, Wang S, Sun K, Chen R, Li L, Wei W, He J. Cancer incidence and mortality in China, 2016. Journal of the National Cancer Center, 2022, 2(1): 1-9. https://doi.org/10.1016/j.jncc.2022.02.002

1. Zeng H, Chen W, Zheng R, et al. Changing cancer survival in China during 2003-15: a pooled analysis of 17 population-based cancer registries. Lancet Glob Health. 2018;6(5):e555-e567. doi:10.1016/S2214-109X(18)30127-X

Malvia S, Bagadi SA, Dubey US, Saxena S. Epidemiology of breast cancer in Indian women. Asia Pac J Clin Oncol. 2017 Aug;13(4):289-295. doi: 10.1111/ajco.12661. Epub 2017 Feb 9. PMID: 28181405.

Mathur P, Sathishkumar K, Chaturvedi M, et al. Cancer Statistics, 2020: Report from National Cancer Registry Programme, India. JCO Glob Oncol. 2020;6:1063-1075. doi:10.1200/GO.20.00122

Indonesia Cancer Registry 2013-2017. National Cancer Center Indonesia, Dharmais Cancer Hospital, Jakarta, Indonesia. 2023. (unpublished)

Allemani C, Weir HK, Carreira H, et al. Global surveillance of cancer survival 1995-2009: analysis of individual data for 25,676,887 patients from 279 population-based registries in 67 countries (CONCORD-2) [published correction appears in Lancet. 2015 Mar 14;385(9972):946]. *Lancet*. 2015;385(9972):977-1010. doi:10.1016/S0140-6736(14)62038-9

Aryannejad A, Saeedi Moghaddam S, Mashinchi B, et al. National and subnational burden of female and male breast cancer and risk factors in Iran from 1990 to 2019: results from the Global Burden of Disease study 2019. Breast Cancer Res. 2023;25(1):47. Published 2023 Apr 26. doi:10.1186/s13058-023-01633-4

Abedi G, Janbabai G, Moosazadeh M, Farshidi F, Amiri M, Khosravi A. Survival Rate of Breast Cancer in Iran: A Meta-Analysis. Asian Pac J Cancer Prev. 2016;17(10):4615-4621. Published 2016 Oct 1. doi:10.22034/apjcp.2016.17.10.4615

Center for Cancer Control and Information Services. National Cancer Center, Japan. Available from <https://ganjoho.jp/reg_stat/statistics/stat/cancer/14_breast.html#anchor1>. Accessed 8 July 2023

1. Kang MJ, Jung KW, Bang SH, et al. Cancer Statistics in Korea: Incidence, Mortality, Survival, and Prevalence in 2020. Cancer Res Treat. 2023;10.4143/crt.2023.447. doi:10.4143/crt.2023.447
2. Malaysia National Cancer Registry Report 2012-2016. National Cancer Institute, Ministry of Health Malaysia. 2019 Available at. <https://nci.moh.gov.my/index.php/ms/main-menu-2/laporan>
3. National Cancer Registry, National Cancer Institute, Ministry of Health Malaysia (2018). Malaysian Study on Cancer Survival (MySCan).

Mongolia cancer registry 2020. Extracted from <http://www.cancer-center.gov.mn/about-us/departments/burtgeltandalt/> [Accessed 13 June 2022]

Shwe KM, Win SM, Thant AN, Htay AMS, Han HM, Paw NHK. Cancer Incidence and Mortality in Central Myanmar: Report of Nay Pyi Taw Population-Based Cancer Registry. Asian Pac J Cancer Prev. 2022 Jan 1;23(1):311-318. doi: 10.31557/APJCP.2022.23.1.311. PMID: 35092400; PMCID: PMC9258644.

Cancer Statistic Report (2013-2017), Nay Pyi Taw Cancer Registry, Department of Medical Services, Ministry of Health, Myanmar. [https://www.mohs.gov.mm/Main/content/publication/cancer-statistics-report-2013-2017-nay-pyi-taw-union-territory-october-2022 Accessed 9 August 2023](https://www.mohs.gov.mm/Main/content/publication/cancer-statistics-report-2013-2017-nay-pyi-taw-union-territory-october-2022%20Accessed%209%20August%202023).

Allemani C, Matsuda T, Di Carlo V, Harewood R, Matz M, Niksic M, et al. Global surveillance of trends in cancer survival 2000-14 (CONCORD-3): analysis of individual records for 37 513 025 patients diagnosed with one of 18 cancers from 322 population-based registries in 71 countries. Lancet. 2018;391(10125):1023-75.

Giri M, Giri M, Thapa RJ, Upreti B, Pariyar B. Breast Cancer in Nepal: Current status and future directions. *Biomed Rep*. 2018;8(4):325-329. doi:10.3892/br.2018.1057

Ghoncheh M, Mohammadian-Hafshejani A, Salehiniya H. Incidence and Mortality of Breast Cancer and their Relationship to Development in Asia. *Asian Pac J Cancer Prev*. 2015;16(14):6081-6087. doi:10.7314/apjcp.2015.16.14.6081

Youlden DR, Cramb SM, Dunn NA, Muller JM, Pyke CM, Baade PD. The descriptive epidemiology of female breast cancer: an international comparison of screening, incidence, survival and mortality. Cancer Epidemiol. 2012;36(3):237-248. doi:10.1016/j.canep.2012.02.007

1. National Registry of Diseases Office 23 Dec 2022. Singapore Cancer Registry Annual Report 2020. https://nrdo.gov.sg/docs/librariesprovider3/default-document-library/scr-2020-annual-report_web-release.pdf?sfvrsn=e0a73b99_0. Accessed 17 March 2022.

Cancer Incidence and Mortality Data, Sri Lanka, 2020 Ministry of Health Sri Lanka. National Cancer Control Programme. https://www.nccp.health.gov.lk/en/question Accessed 10 June 2023.

1. Registrar General’s department statistics (2019) Department of Census and Statistics, Sri Lanka. <http://www.statistics.gov.lk/Population/Vital_Statistics-2019> Accessed 5 July 2023.

Balawardena J, Skandarajah T, Rathnayake W, Joseph N. Breast Cancer Survival in Sri Lanka. JCO Glob Oncol. 2020;6:589-599. doi:10.1200/JGO.20.00003

Rojanamatin J, Ukranun W, Supaatagorn P, Chiawiriyabunya I, Wongsena W, Chiawerawattana A, et al. Cancer in Thailand Vol. X 2016-2018. Bangkok, Thailand: Ministry of Public Health, National Cancer Institute, 2021.

Allemani C, Matsuda T, Di Carlo V, Harewood R, Matz M, Niksic M, et al. Global surveillance of trends in cancer survival 2000-14 (CONCORD-3): analysis of individual records for 37 513 025 patients diagnosed with one of 18 cancers from 322 population-based registries in 71 countries. Lancet. 2018;391(10125):1023-75.

1. Lan NH, Laohasiriwong W, Stewart JF. Survival probability and prognostic factors for breast cancer patients in Vietnam. Glob Health Action. 2013 Jan 17;6:1-9. doi: 10.3402/gha.v6i0.18860. PMID: 23336619; PMCID: PMC3549066.

**For data in Table 2:**

1. WHO Global Health Observatory Data Repository database 2021. Existence of national screening program for breast cancer. Available at https://www.who.int/data/gho/data/indicators/indicator-details/GHO/existence-of-national-screening-program-for-breast-cancer. Accessed 30 April 2023.
2. Tracking Universal Health Coverage: 2021 global monitoring report. Geneva: World Health Organization and

International Bank for Reconstruction and Development / The World Bank; 2021. Licence: CC BY-NC-SA 3.0 IGO. Accessed 16 May 2023.

1. Ministry of Health and Family Welfare, Government of Bangladesh. National Health Information System (DHIS2) Available from [https://centraldhis.mohfw.gov.bd/dhismohfw/dhis-webcommons/security/login.action Accessed 17 August 2023](https://centraldhis.mohfw.gov.bd/dhismohfw/dhis-webcommons/security/login.action%20Accessed%2017%20August%202023)
2. Country fact sheet: Bangladesh. Breast cancer screening programme. Available at https://canscreen5.iarc.fr/?page=countryfactsheetbreast&q=BGD&rc= Accessed on 3 July 2023.
3. World Health organization. WHO supports early detection and control of cervical and breast cancer in Bangladesh 2020, 10 Nov. Available from: https://www.who.int/bangladesh/news/detail/10-11-2020-who-supports-early-detection-and-control-of-cervical-and-breast-cancer-in-bangladesh.
4. Story HL, Love RR, Salim R, Roberto AJ, Krieger JL, Ginsburg OM. Improving outcomes from breast cancer in a low-income country: Lessons from Bangladesh. Int J Breast Cancer. 2012;2012:423562. doi: 10.1155/2012/423562. Epub 2011 Dec 5. PMID: 22295245; PMCID: PMC3262600.
5. Ministry of Health Bhutan. Guideline For Screening Gastric Cancer, Cervical Cancer & Breast Cancer Available at https://www.moh.gov.bt/wp-content/uploads/ict-files/2021/06/GUIDELINE-FOR-SCREENING-GASTRIC-CERVICAL-AND-BRTEAST-CANCERS.pdf Accessed 23 March 2023
6. National Health Screening Guidelines on Noncommunicable Diseases 2020. Noncommunicable Disease (NCD) Prevention Unit, Ministry of Health Brunei Darussalam. https://www.moh.gov.bn/Shared%20Documents/MOH_National%20Health%20Screening%20Guideline%20on%20NCDs_23%20Jul%202020.pdf
7. Suhaimi, A.M.A.; Abdul Rahman, H.; Ong, S.K.; Koh, D. Predictors of non-communicable diseases screening behaviours among adult population in Brunei Darussalam: a retrospective study. Journal of Public Health 2021 29, 1303-1312.
8. Brunei Darussalam Cancer Registry Report 2002 – 2021 (2022). Noncommunicable Disease (NCD) Prevention Unit, Ministry of Health Brunei Darussalam. https://www.moh.gov.bn/Shared%20Documents/DOWNLOADS/BDCR%202002-2021%20vMay2023.pdf
9. Ley P, Hong C, Varughese J, Camp L, Bouy S, Maling E. Challenges in the Management of Breast Cancer in a Low Resource Setting in South East Asia. Asian Pac J Cancer Prev. 2016;17(7):3459-63. PMID: 27509992.
10. Breast cancer screening guideline for Chinese women. Cancer Biol Med. 2019;16(4):822-824. doi:10.20892/j.issn.2095-3941.2019.0321
11. Mi ZH, Ren JS, Zhang HZ, et al. Zhonghua Yu Fang Yi Xue Za Zhi. 2016;50(10):887-892. doi:10.3760/cma.j.issn.0253-9624.2016.10.010
12. Sun L, Legood R, Sadique Z, Dos-Santos-Silva I, Yang L. Cost-effectiveness of risk-based breast cancer screening programme, China. Bull World Health Organ. 2018;96(8):568-577. doi:10.2471/BLT.18.207944
13. Mei Zhang, Heling Bao, Xiao Zhang, Zhengjing Huang, Zhenping Zhao, Chun Li, Maigeng Zhou, Jing Wu, Limin Wang, Linhong Wang. Breast Cancer Screening Coverage — China, 2018–2019[J]. China CDC Weekly, 2023, 5(15): 321-326. doi: 10.46234/ccdcw2023.062
14. Zeng H, Ran X, An L, Zheng R, Zhang S, Ji J, Zhang Y, Chen W, Wei W, He J. Disparities in stage at diagnosis for five common cancers in China: a multicentre, hospital-based, observational study. Lancet Public Health 2021 6:e877-87.
15. An J, Hershberger PE, Ferrans CE. Delayed Presentation, Diagnosis, and Treatment of Breast Cancer Among Chinese Women: An Integrative Literature Review. Cancer Nurs. 2023;46(3):217-232. doi:10.1097/NCC.0000000000001074
16. Li YL, Qin YC, Tang LY, et al. Patient and Care Delays of Breast Cancer in China. Cancer Res Treat. 2019;51(3):1098-1106. doi:10.4143/crt.2018.386
17. Indian Council of Medical Research. Consensus document for management of breast cancer. 2016. <https://main.icmr.nic.in/sites/default/files/guidelines/Breast_Cancer.pdf> [Accessed 12 July 2023]
18. Meena S, Rathore M, Gupta A, Kumawat P, Singh A. Assessment of National Program for Prevention and Control of Cancer, Diabetes, CVD and Stroke (NPCDCS): An observational study in rural Jaipur, Rajasthan. J Family Med Prim Care. 2022;11(7):3667-3672. doi:10.4103/jfmpc.jfmpc_2281_21
19. Mehrotra R, Yadav K. Cervical Cancer: Formulation and Implementation of Govt of India Guidelines for Screening and Management. Indian J Gynecol Oncol. 2022;20(1):4. doi: 10.1007/s40944-021-00602-z. Epub 2021 Dec 27. PMID: 34977333; PMCID: PMC8711687.
20. Ministry of Health and Family Welfare. Operational Framework: Management of common cancers. 2016. <https://main.mohfw.gov.in/sites/default/files/Operational%20Framework%20Management%20of%20Common%20Cancers_1.pdf> [Accessed 12 July 2023]
21. Mittra I, Mishra GA, Dikshit RP, et al. Effect of screening by clinical breast examination on breast cancer incidence and mortality after 20 years: prospective, cluster randomised controlled trial in Mumbai BMJ. 2021;372:n256. Published 2021 Feb 24. doi:10.1136/bmj.n256
22. Negi J, Nambiar D. Intersectional social-economic inequalities in breast cancer screening in India: analysis of the National Family Health Survey. BMC Womens Health. 2021;21(1):324. Published 2021 Sep 7. doi:10.1186/s12905-021-01464-5
23. Mathur P, Sathishkumar K, Chaturvedi M, et al. Cancer Statistics, 2020: Report from National Cancer Registry Programme, India. JCO Glob Oncol. 2020;6:1063-1075. doi:10.1200/GO.20.00122
24. Somanna SN, Nandagudi Srinivasa M, Chaluvarayaswamy R, Malila N. Time Interval between Self-Detection of Symptoms to Treatment of Breast Cancer. Asian Pac J Cancer Prev. 2020;21(1):169-174. Published 2020 Jan 1. doi:10.31557/APJCP.2020.21.1.169
25. Kumar A, Bhagabaty SM, Tripathy JP, Selvaraj K, Purkayastha J, Singh R. Delays in Diagnosis and Treatment of Breast Cancer and the Pathways of Care: A Mixed Methods Study from a Tertiary Cancer Centre in North East India. Asian Pac J Cancer Prev. 2019 Dec 1;20(12):3711-3721. doi: 10.31557/APJCP.2019.20.12.3711. PMID: 31870113; PMCID: PMC7173377.
26. Census 2020 Indonesia (unpublished data)
27. Indonesia Cancer Registry 2013-2017 (unpublished data)
28. Hutajulu SH, Prabandari YS, Bintoro BS, et al. Delays in the presentation and diagnosis of women with breast cancer in Yogyakarta, Indonesia: A retrospective observational study. PLoS One. 2022;17(1):e0262468. doi:10.1371/journal.pone.0262468
29. Hospital Based Cancer Registry of Dharmais NCC 2018 (unpublished data)
30. Badakhsh M, Balouchi A, Taheri S, Bouya S, Ahmadidarehsima S, Aminifard M. Attitude and Practice Regarding Breast Cancer Early Detection among Iranian Women: A Systematic Review. Asian Pac J Cancer Prev. 2018;19(1):9-16. doi:10.22034/APJCP.2018.19.1.9
31. Foroozani E, Ghiasvand R, Mohammadianpanah M, et al. Determinants of delay in diagnosis and end stage at presentation among breast cancer patients in Iran: a multi-center study. Sci Rep. 2020;10(1):21477. doi:10.1038/s41598-020-78517-6
32. Comprehensive survey of living conditions, Ministry of Health, Labour and Welfare, Japan
33. Annual Report of Hospital-Based Cancer Registries 2021. (2023). Cancer Information Service, National Cancer Center, Japan
34. Song SY, Lee YY, Shin HY, et al. Trends in breast cancer screening rates among Korean women: results from the Korean National Cancer Screening Survey, 2005-2020. Epidemiol Health. 2022;44:e2022111. doi:10.4178/epih.e2022111
35. Choi JE, Kim Z, Park CS, the Korean Breast Cancer Society et al. Breast Cancer Statistics in Korea, 2019. J Breast Cancer. 2023;26:e27. https://doi.org/10.4048/jbc.2023.26.e27
36. Shin DW, Cho J, Kim SY, et al. Delay to curative surgery greater than 12 weeks is associated with increased mortality in patients with colorectal and breast cancer but not lung or thyroid cancer. Ann Surg Oncol. 2013;20(8):2468-2476. doi:10.1245/s10434-013-2957-y
37. Luangxay T, Virachith S, Hando K, et al. Subtypes of Breast Cancer in Lao P.D.R.: A Study in a Limited-Resource Setting. Asian Pac J Cancer Prev. 2019;20(2):589-594. Published 2019 Feb 26. doi:10.31557/APJCP.2019.20.2.589
38. Htay MNN, Donnelly M, Schliemann D, et al. Breast Cancer Screening in Malaysia: A Policy Review. Asian Pac J Cancer Prev. 2021;22(6):1685-1693. Published 2021 Jun 1. doi:10.31557/APJCP.2021.22.6.1685
39. Management of Breast Cancer (3rd Edition) Clinical Practice Guidelines 2019. Ministry of Health Malaysia.
40. Yip CH, Bhoo Pathy N, Teo SH. A review of breast cancer research in Malaysia. Med J Malaysia. 2014;69 Suppl A:8-22.
41. Malaysia National Cancer Registry Report 2012-2016. National Cancer Institute, Ministry of Health Malaysia. 2019 Available at. https://nci.moh.gov.my/index.php/ms/main-menu-2/laporan
42. Mohd Mujar NM, Dahlui M, Emran NA, et al. Breast Cancer Care Timeliness Framework: A Quality Framework for Cancer Control. JCO Glob Oncol. 2022;8:e2100250. doi:10.1200/GO.21.00250
43. Norsa'adah B, Rampal KG, Rahmah MA, Naing NN, Biswal BM. Diagnosis delay of breast cancer and its associated factors in Malaysian women. BMC Cancer. 2011;11:141. Published 2011 Apr 17. doi:10.1186/1471-2407-11-141
44. Lim GC, Aina EN, Cheah SK, Ismail F, Ho GF, Tho LM, et al. Closing the global cancer divide- performance of breast cancer care services in a middle income developing country. BMC Cancer. 2014; 14: 212.
45. Ministry of Health Mongolia 2023. The national breast cancer screening report. (unpublished)
46. Mongolia cancer registry 2020. Extracted from http://www.cancer-center.gov.mn/about-us/departments/burtgeltandalt/ [Accessed 13 June 2022]
47. Moh Myint, Nyein Moh, Nursalam Nursalam, and Eka Mishbahatul Mar’ah Has. "Exploring the Influencing Factors on Breast Self-Examination Among Myanmar Women: A Qualitative Study." (2020).
48. Dhakal R, Noula M, Roupa Z, Yamasaki EN. A Scoping Review on the Status of Female Breast Cancer in Asia with a Special Focus on Nepal. Breast Cancer (Dove Med Press). 2022;14:229-246. Published 2022 Aug 26. doi:10.2147/BCTT.S366530
49. Bhandari D, Shibanuma A, Kiriya J, Hirachan S, Ong KIC, Jimba M. Factors associated with breast cancer screening intention in Kathmandu Valley, Nepal. PLoS One. 2021;16(1):e0245856. Published 2021 Jan 22. doi:10.1371/journal.pone.0245856
50. Baral S, Silwal SR, Shrestha UM, Lamichhane D. Evaluation of Quality Indicators of Breast Cancer Management at a Tertiary Cancer Center in Nepal. JCO Glob Oncol. 2022;8:e2100303. doi:10.1200/GO.21.00303
51. Talib, Z., Amersi, F., Harit, A. et al. Promoting Breast Cancer Awareness and Clinical Breast Examination in the LMIC: Experiences from Tajikistan, Pakistan and Kenya. Curr Breast Cancer Rep 11, 152–157 (2019). https://doi.org/10.1007/s12609-019-00321-7
52. Shamsi U, Khan S, Azam I, et al. Patient Delay in Breast Cancer Diagnosis in Two Hospitals in Karachi, Pakistan: Preventive and Life-Saving Measures Needed. JCO Glob Oncol. 2020;6:873-883. doi:10.1200/GO.20.00034
53. Khokher S, Qureshi MU, Riaz M, Akhtar N, Saleem A. Clinicopathologic profile of breast cancer patients in Pakistan: ten years data of a local cancer hospital. Asian Pac J Cancer Prev. 2012;13(2):693-698. doi:10.7314/apjcp.2012.13.2.693
54. Wu TY, Lee J. Promoting Breast Cancer Awareness and Screening Practices for Early Detection in Low-Resource Settings. Eur J Breast Health. 2018;15(1):18-25. Published 2018 Nov 21. doi:10.5152/ejbh.2018.4305
55. Lim YX, Lim ZL, Ho PJ, Li J. Breast Cancer in Asia: Incidence, Mortality, Early Detection, Mammography Programs, and Risk-Based Screening Initiatives. Cancers (Basel). 2022 Aug 30;14(17):4218. doi: 10.3390/cancers14174218. PMID: 36077752; PMCID: PMC9454998.
56. National Population Health Survey 2022. Epidemiology & Disease Control Division and Policy, Research & Surveillance Group. Ministry of Health and Health Promotion Board, Singapore.
57. National Registry of Diseases Office 23 Dec 2022. Singapore Cancer Registry Annual Report 2020. https://nrdo.gov.sg/docs/librariesprovider3/default-document-library/scr-2020-annual-report_web-release.pdf?sfvrsn=e0a73b99_0. Accessed 17 March 2022.
58. Joint Breast Cancer Registry (query date: 26.05.2023) Available at https://www.nccs.com.sg/research-innovation/pages/joint-breast-cancer-registry.aspx
59. Wong FY, Wong RX, Zhou S, et al. Effects of housing value and medical subsidy on treatment and outcomes of breast cancer patients in Singapore: A retrospective cohort study. Lancet Reg Health West Pac. 2020;6:100065. Published 2020 Dec 8. doi:10.1016/j.lanwpc.2020.100065
60. Sri Lanka STEPS Survey 2021. Fact Sheet. STEPS Survey 2021. The STEPS survey of noncommunicable disease (NCD) risk factors. https://www.ncd.health.gov.lk/images/pdf/Steps-Fact-Sheet.pdf Accessed 5 July 2023.
61. Wijeratne DT, Gunasekera S, Booth CM, et al. Demographic, tumour, and treatment characteristics of female patients with breast cancer in Sri Lanka; results from a hospital-based cancer registry. BMC Cancer. 2021;21(1):1175. Published 2021 Nov 3. doi:10.1186/s12885-021-08929-8
62. Country fact sheet: Thailand. Breast cancer screening programme. Available at https://canscreen5.iarc.fr/?page=countryfactsheetbreast&q=THA&rc= Accessed 5 July 2023
63. Srithamrongsawat S, Aekplakorn W, Jongudomsuk P, Thammatach-aree J, Patcharanarumol W, Swasdiworn W, et al. Funding health promotion and prevention - the Thai experience. 2010.
64. Mukem S, Sriplung H, McNeil E, Tangcharoensathien V. Breast cancer screening among women in Thailand: analyses of population-based household surveys. J Med Assoc Thai. 2014 Nov;97(11):1106-18. PMID: 25675674.
65. Thaineua V, Ansusinha T, Auamkul N, Taneepanichskul S, Urairoekkun C, Jongvanich J, et al. Impact of regular Breast Self-Examination on breast cancer size, stage, and mortality in Thailand. Breast J. 2020;26(4):822-4.
66. Thai Cancer Base data. Ministry of Public Health, Bangkok, Thailand 2023 Available at https://canceranywhere.com:8081/tcb2023/#/user/login-v3 (in Thai) Accessed September 7, 2023.
67. Ministry of Health Vietnam. Guidelines for prevention, screening, early detection and management of breast and cervical cancer in the community under Project 818 to 2030. Available at https://emohbackup.moh.gov.vn/publish/home?documentId=8279 (Vietnamese)
68. Ngan TT, Jenkins C, Minh HV, Donnelly M, O'Neill C. Breast cancer screening practices among Vietnamese women and factors associated with clinical breast examination uptake. PLoS One. 2022;17(5):e0269228. doi:10.1371/journal.pone.0269228
69. Duc NB. Breast cancer situation in women in some provinces/cities from 2001 to 2007 [in Vietnamese]. Vietnam J Oncol. 2009;1:5–11.
70. Nguyen SM, Nguyen QT, Nguyen LM, et al. Delay in the diagnosis and treatment of breast cancer in Vietnam. Cancer Med. 2021;10(21):7683-7691. doi:10.1002/cam4.4244
