# Supplementary material for: Feasibility of monitoring Global Breast Cancer Initiative Framework key performance indicators in 21 Asian National Cancer Centers Alliance member countries
Source: eClinicalMedicine. 2023 Dec 16;67:102365. doi: 10.1016/j.eclinm.2023.102365 (PMC10731600; doi:10.1016/j.eclinm.2023.102365)
Supplement: Supplementary Appendix B [file mmc2.docx]

**Appendix B Standardised data collection form and questionnaire**

Standardised questions for data collection on breast cancer indicators, breast cancer screening, and barriers in each ANCCA member country:

1. To provide the latest published statistics and references in your country on breast cancer indicators. If the national statistic were unavailable, published regional or institutional statistics from the hospitals or cancer centres to be presented:

- Age-standardised incidence rate (ASIR) per 100,000 women
- Age-standardised mortality rate (ASMR) per 100,000 women
- Percentage of breast cancer patients with a 5-year survival rate (%)
- Percentage of eligible aged women screened for breast cancer (%)
- Percentage of breast cancer diagnosed in the early stages (%)
- Percentage of patients diagnosed within 60 days of initial presentation (%)
- Time interval between first presentation and diagnosis of breast cancer (days)
- Time interval between diagnosis and starting breast cancer treatment (days)
- Percentage of patients with breast cancer completed multidisciplinary treatment (%)

1. To select statements that are correct or applicable to breast cancer control in your country:

- There is national health held regularly campaign to raise awareness of breast cancer prevention and early detection in the country.
- There is a national screening programme for breast cancer in the country.
- Breast cancer detection and treatment are affordable or subsidised in the country.
- There is a shortage of healthcare professionals trained or skilled in the early detection and management of breast cancer.
- There is inadequate access to or a shortage of healthcare facilities for screening, diagnosis,and treatment of breast cancer, either in urban or rural areas.
- National surveillance data on breast cancer are available in the country.
- There are disparities in health service accessibility and utilisation across different social groups in the country.
- There is inadequate multi-stakeholder involvement in breast cancer prevention (e.g., community healthcare workers, NGOs, patient support groups).
- Pre-treatment diagnosis of breast cancer using biopsy is the usual practice in the country.
- Pre-treatment ImmunoHistoChemistry (e.g., ER, PR, HER2 receptors) testing on breast cancer biopsies is provided.
- Women may be concerned or hesitant to go for breast cancer screening or treatment due to the lack of awareness, knowledge, psychological, or cultural reasons.

List any country-specific barriers to breast cancer control in your country.
