# Supplementary material for: Feasibility of monitoring Global Breast Cancer Initiative Framework key performance indicators in 21 Asian National Cancer Centers Alliance member countries
Source: eClinicalMedicine. 2023 Dec 16;67:102365. doi: 10.1016/j.eclinm.2023.102365 (PMC10731600; doi:10.1016/j.eclinm.2023.102365)
Supplement: Supplementary Appendix C — Fig 4: a) The dotted (regression) line represents the linear regression model (y = 0.0312x + 59.262) that best fit the shown countries’ proportion of patients diagnosed in stages I and II (Pillar 1 KPI) and local registry Age Standardised Mortality Rate (ASMR) of breast cancer per 100,000 female population, indicated by the blue dots. The graph shows that there is a positive linear relationship between proportion of patients diagnosed in Stage I and II (Pillar 1 KPI) and local registry’s ASMR as the value of one attribute increases, the other remain almost constant. The R2 value of 3E-05 indicates a very weak relationship. b) The dotted (regression) line represents the linear regression model (y = 0.535x + 43.04) that best fit the shown countries’ proportion of patients diagnosed in Stage I and II (Pillar 1 KPI) and GLOBOCAN 2020 estimates on Age Standardised Mortality Rate (ASMR) per 100,000 female population, indicated by the blue dots. The graph shows that there is a positive linear relationship between proportion of patients diagnosed in Stage I and II (Pillar 1 KPI) and GLOBOCAN 2020 estimates of ASMR as the value of one attribute increases, so does the other. The R2 value of 0.0117 indicates a very weak relationship. Fig 5: a) The dotted (regression) line represents the linear regression model (y = -1.0712x + 89.407) that best fit the shown countries’ 5-year survival rate and local registry age standardised mortality rate ASMR) per 100,000 female population, indicated by the blue dots. The graph shows that there is a negative linear relationship between 5-year survival rate and local registry’s ASMR per 100,000 female population as the value of one attribute increases, the other decreases. The R2 value of 0.2202 indicates a very moderate relationship. b) The dotted (regression) line represents the linear regression model (y = -0.3294x + 76.786) that best fit the shown countries’ Universal Health Coverage index and the age standardised mortality rate (ASMR) per [file mmc3.pptx]

## Slide 1
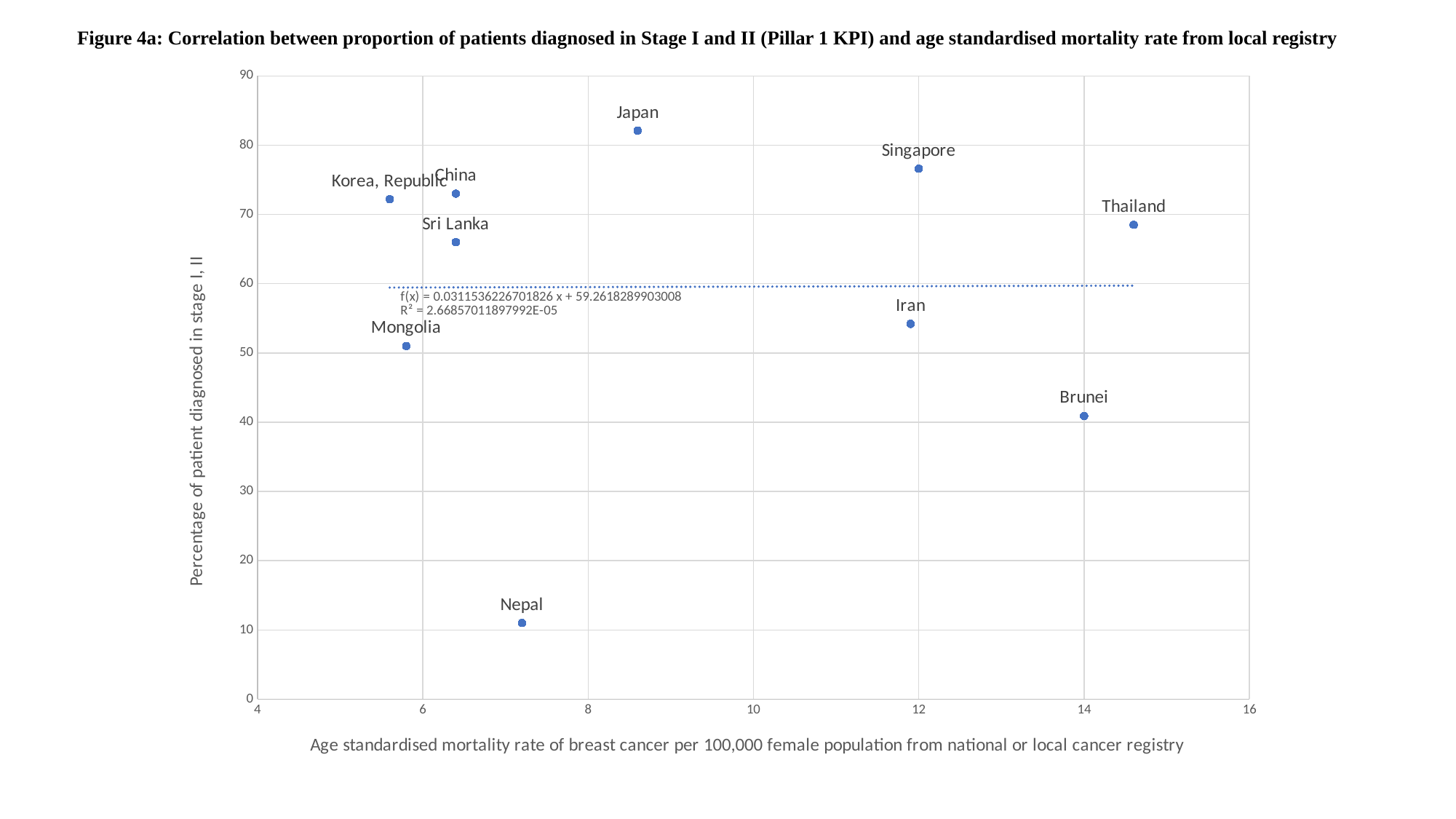

Figure 4a: Correlation between proportion of patients diagnosed in Stage I and II (Pillar 1 KPI) and age standardised mortality rate from local registry
### Chart
| Category | % of patient diagnosed in stage I, II |
|---|---|

## Slide 2
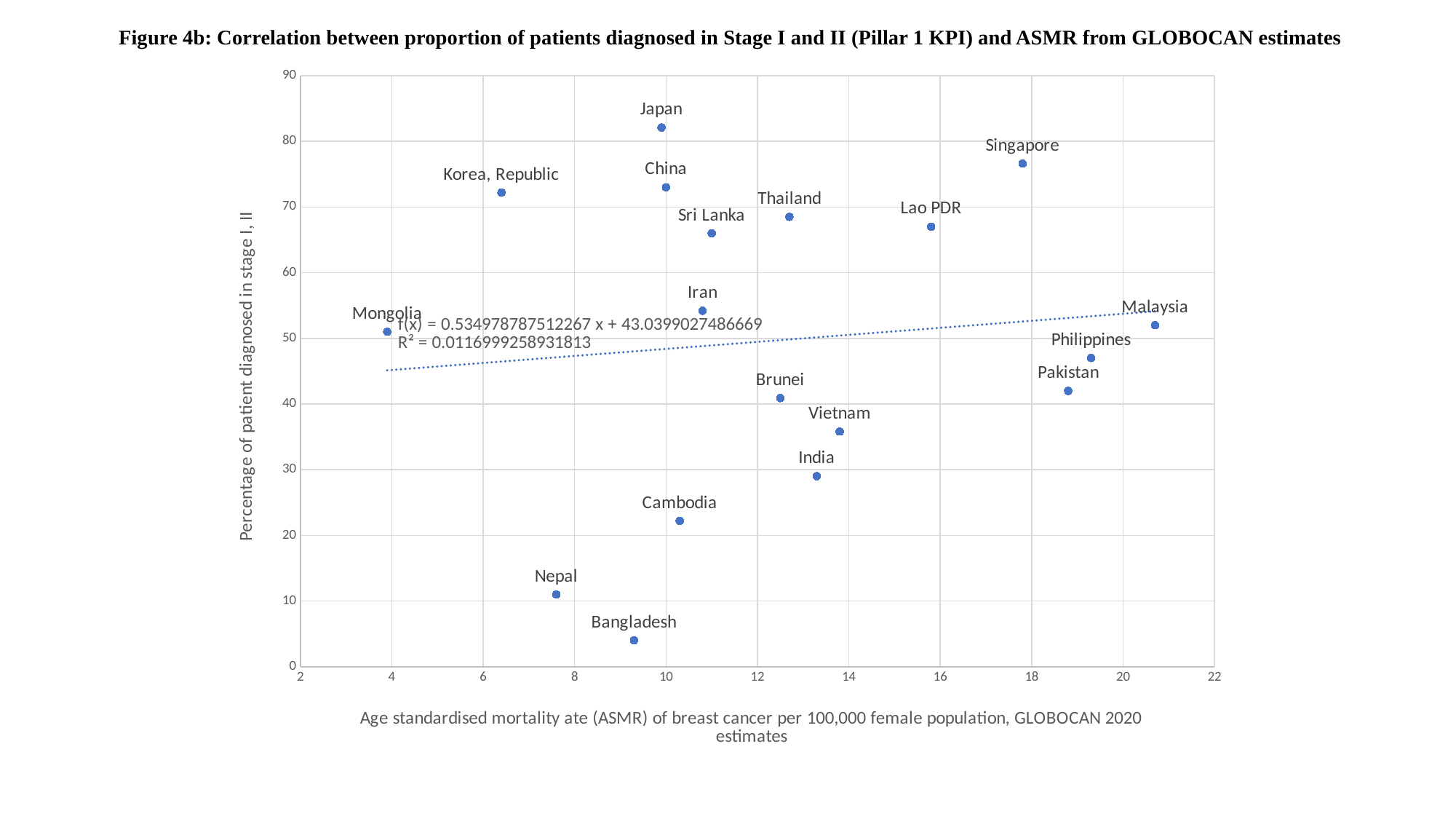

Figure 4b: Correlation between proportion of patients diagnosed in Stage I and II (Pillar 1 KPI) and ASMR from GLOBOCAN estimates
### Chart
| Category | % of patient diagnosed in stage I, II |
|---|---|

## Slide 3
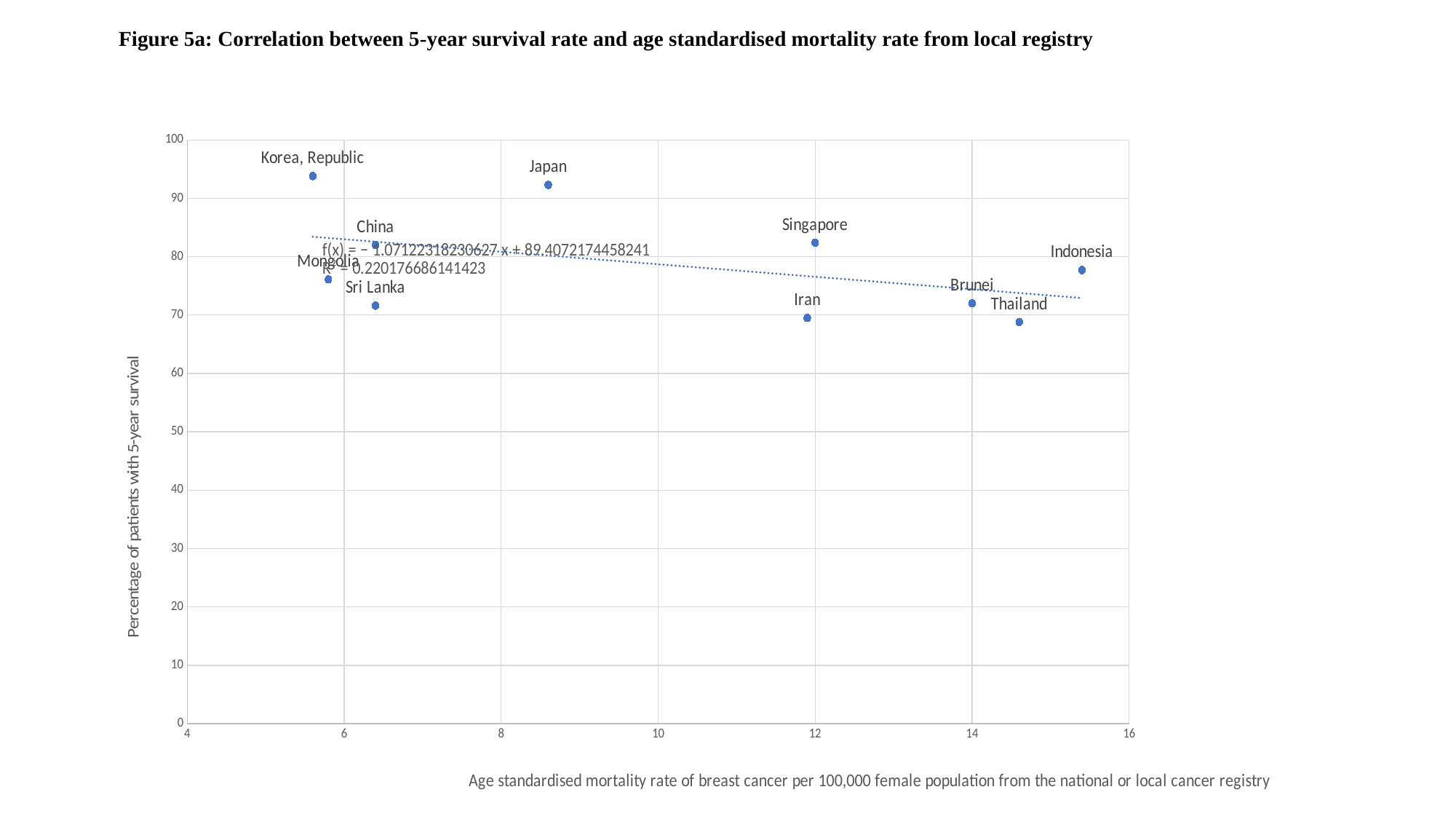

Figure 5a: Correlation between 5-year survival rate and age standardised mortality rate from local registry
### Chart
| Category | % 5-year survival |
|---|---|

## Slide 4
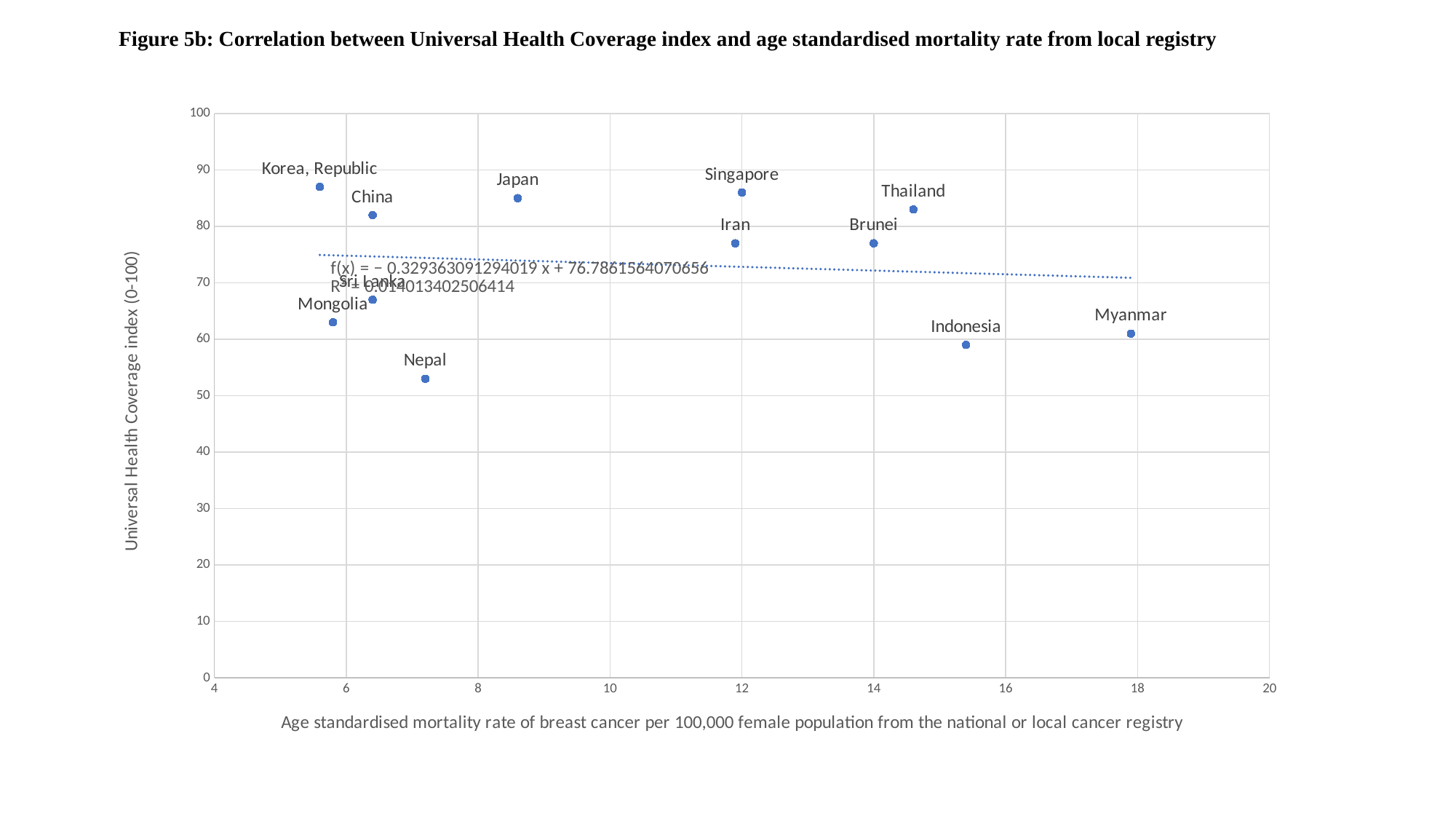

Figure 5b: Correlation between Universal Health Coverage index and age standardised mortality rate from local registry
### Chart
| Category | UHC index (0-100) |
|---|---|

## Slide 5
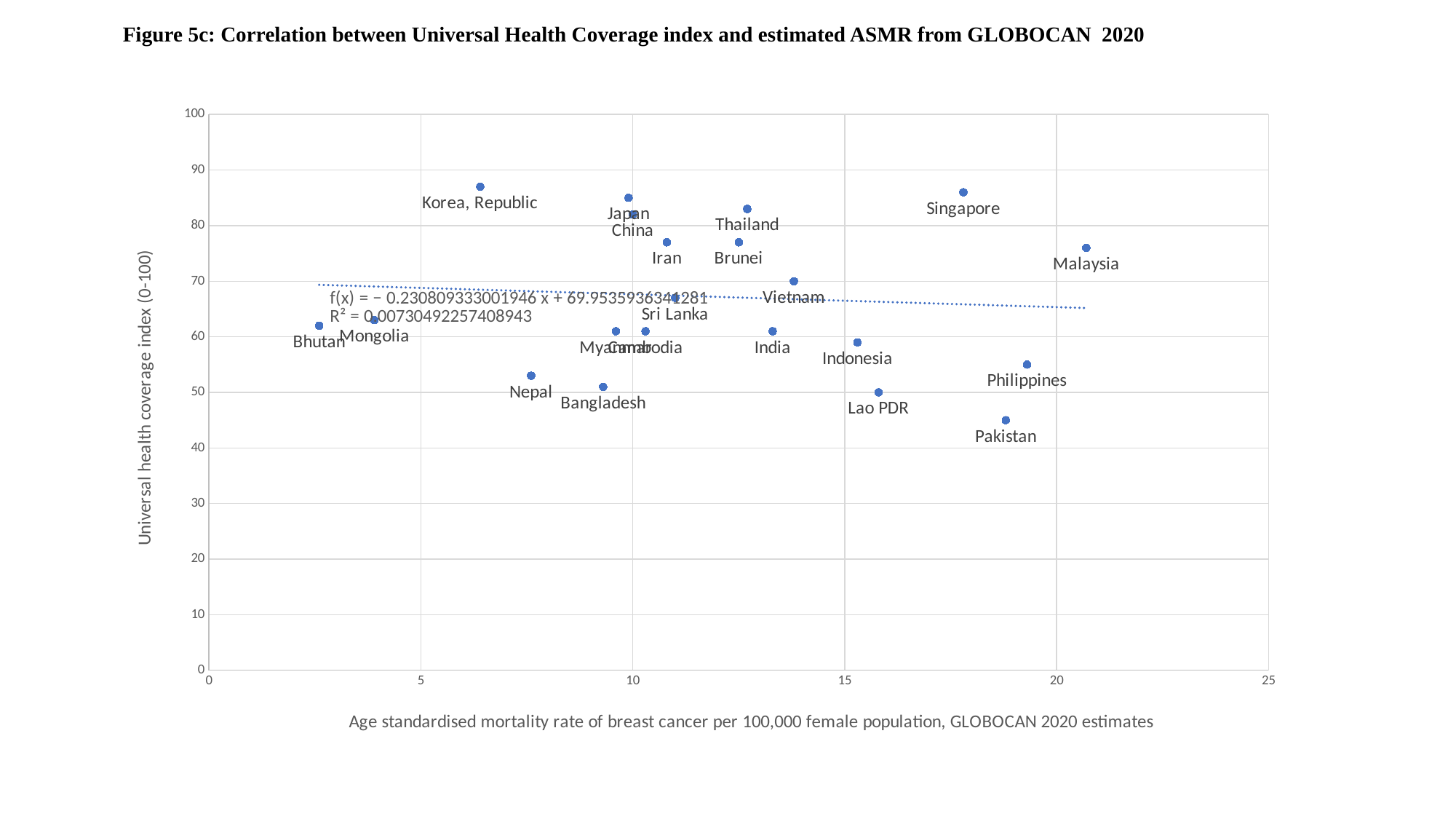

Figure 5c: Correlation between Universal Health Coverage index and estimated ASMR from GLOBOCAN 2020
### Chart
| Category | UHC index (0-100) |
|---|---|

## Slide 6
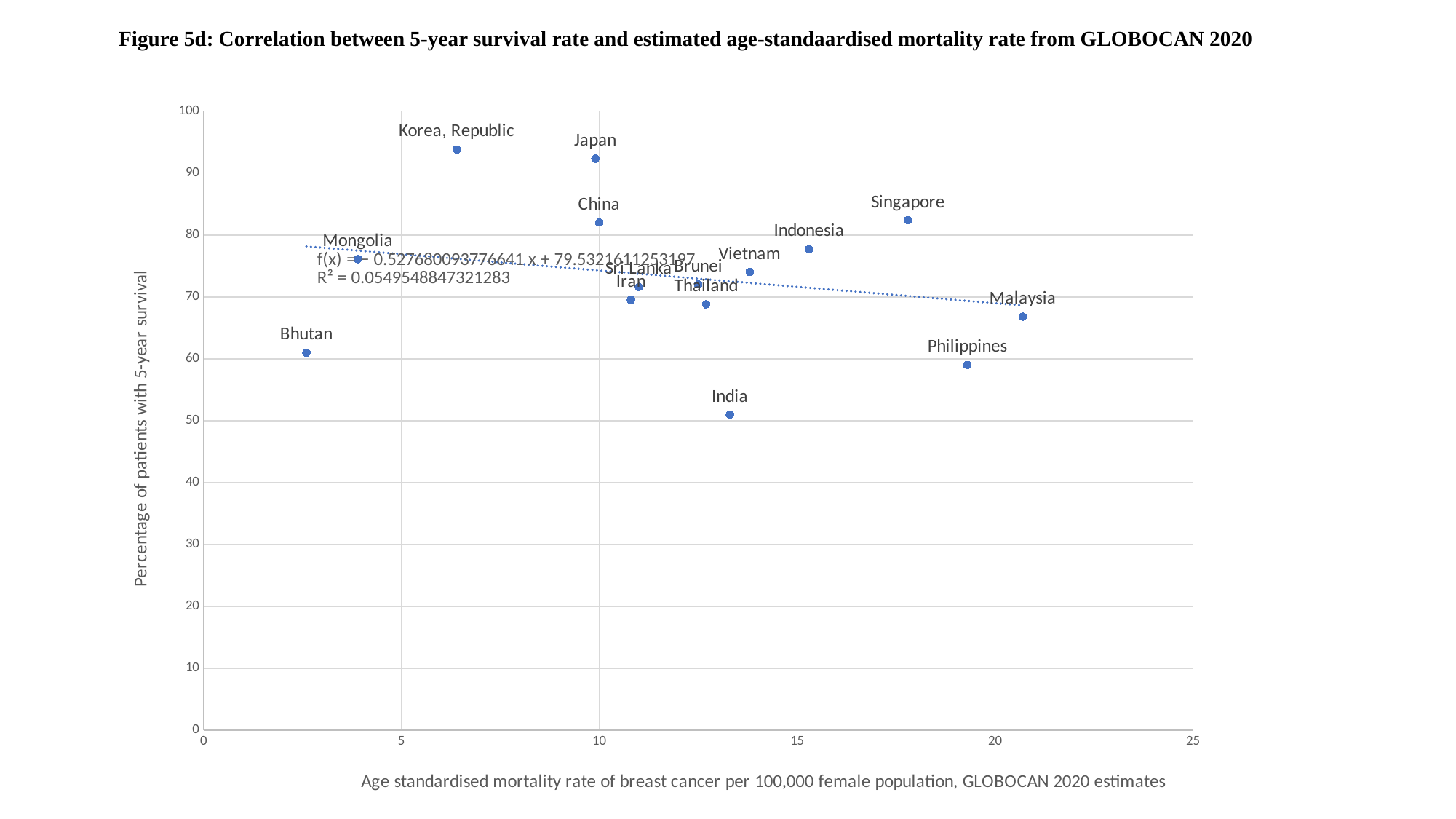

Figure 5d: Correlation between 5-year survival rate and estimated age-standaardised mortality rate from GLOBOCAN 2020
### Chart
| Category | % 5-year survival |
|---|---|
