# Supplementary material for: Feasibility of monitoring Global Breast Cancer Initiative Framework key performance indicators in 21 Asian National Cancer Centers Alliance member countries
Source: eClinicalMedicine. 2023 Dec 16;67:102365. doi: 10.1016/j.eclinm.2023.102365 (PMC10731600; doi:10.1016/j.eclinm.2023.102365)
Supplement: Supplementary Appendix D [file mmc4.docx]

**Appendix D Additional statistical analyses**


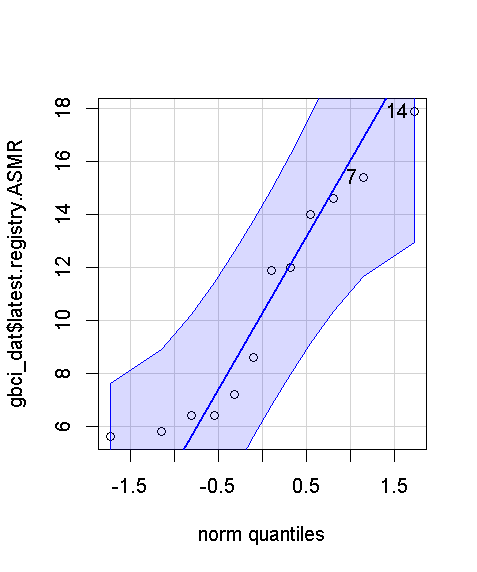
**I. Test for normal distribution**
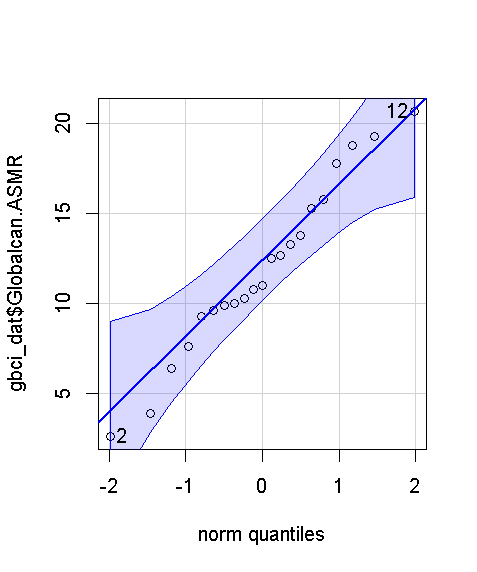


| **variable** | **W** | **p-value** |
| --- | --- | --- |
| Globalcan.ASMR | 0.97633 | 0.8646 |
| local.registry.ASMR | 0.89682 | 0.1443 |
| 5.year.survival | 0.97834 | 0.9569 |
| diagnosed.in.stage.I..II | 0.95185 | 0.4547 |
| UHC.index..0.100 | 0.93853 | 0.2038 |


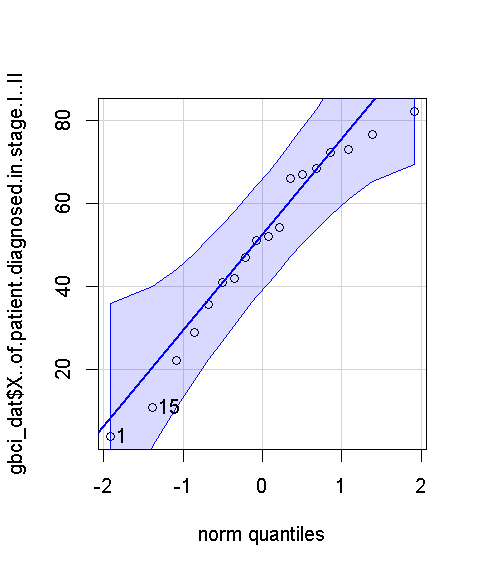

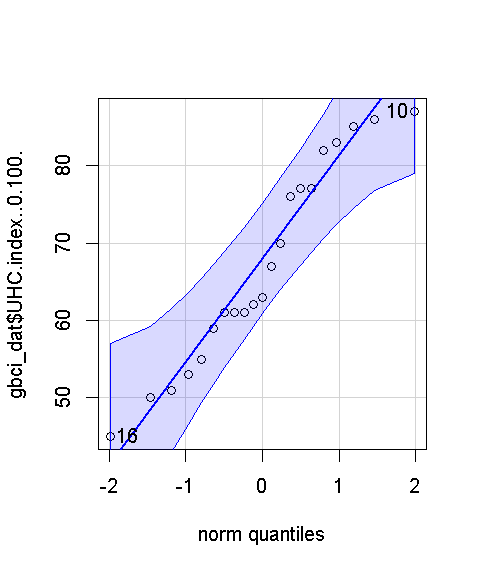

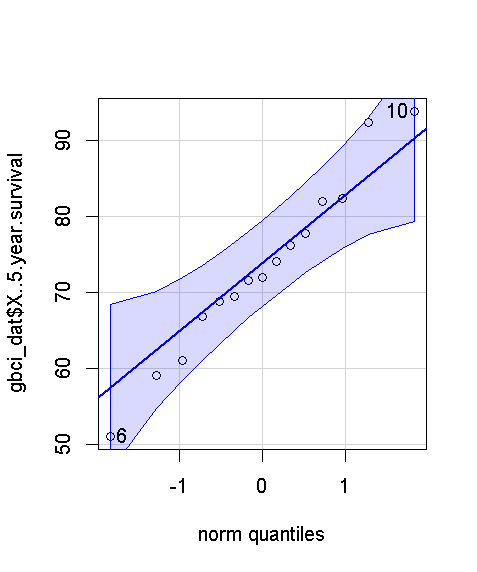


**II.. 95% confidence intervals for correlation coefficients**

|  | **corr.** | **LCI** | **UCI** |
| --- | --- | --- | --- |
| Pillar 1 KPI & UHC index | 0.666 | 0.289 | 0.864 |
| Pillar 1 KPI & 5-year survival | 0.760 | 0.360 | 0.924 |
| Pillar 1 KPI & local registry ASMR | 0.005 | -0.626 | 0.633 |
| Pillar 1 KPI & GLOBOCAN ASMR | 0.108 | -0.378 | 0.547 |
| 5-year survival rate & local registry ASMR | -0.469 | -0.848 | 0.228 |
| UHC index & local registry ASMR | -0.118 | -0.648 | 0.489 |
| UHC index & GLOBOCAN ASMR | -0.085 | -0.499 | 0.359 |
| 5-year survival rate & GLOBOCAN ASMR | -0.234 | -0.667 | 0.316 |

**III. Pearson correlation coefficients, weighted correlations and change in correlation strength following weightage to respective female populations**

|  | **corr.** | **w. corr.** | **change in corr. strength** |
| --- | --- | --- | --- |
| Pillar 1 KPI & UHC index | 0.666 | 0.873 | Strong to very strong |
| Pillar 1 KPI & 5-year survival | 0.760 | 0.966 | Strong to very strong |
| Pillar 1 KPI & local registry ASMR | 0.005 | -0.240 | Very weak to weak |
| Pillar 1 KPI & GLOBOCAN ASMR | 0.108 | -0.413 | Very weak to moderate |
| 5-year survival rate & local registry ASMR | -0.469 | -0.541 | Moderate |
| UHC index & local registry ASMR | -0.118 | -0.767 | Very weak to strong |
| UHC index & GLOBOCAN ASMR | -0.085 | -0.686 | Very weak to strong |
| 5-year survival rate & GLOBOCAN ASMR | -0.234 | -0.604 | Weak to strong |

**IV. Sensitivity analyses with alternative assumptions on missing data**

|  | **Pillar 1 KPI & UHC service coverage index** | | **Pillar 1 KPI & 5-year survival** | | **Pillar 1 KPI & local registry ASMR** | |
| --- | --- | --- | --- | --- | --- | --- |
|  | corr | w. corr | corr | w. corr | corr | w. corr |
| removal | **0.666** | **0.873** | **0.760** | **0.966** | 0.005 | -0.240 |
| replace with zero | 0.631 | 0.840 | 0.529 | 0.616 | 0.045 | 0.167 |
| replace with mean | 0.651 | 0.851 | 0.461 | 0.788 | *-0.544* | *-0.544* |
| MICE | 0.628 | 0.803 | 0.652 | 0.800 | -0.102 | -0.219 |
|  |  |  |  |  |  |  |
|  | **Pillar 1 KPI & GLOBOCAN ASMR** | | **5-year survival rate & local registry ASMR** | | **UHC index & local registry ASMR** | |
|  | corr | w. corr | corr | w. corr | corr | w. corr |
| removal | 0.108 | -0.413 | -0.469 | -0.541 | -0.118 | **-0.767** |
| replace with zero | 0.229 | -0.442 | 0.272 | 0.470 | 0.433 | 0.455 |
| replace with mean | 0.095 | -0.394 | -0.273 | -0.437 | -0.081 | -0.646 |
| MICE | -0.004 | -0.307 | -0.143 | -0.212 | 0.140 | -0.099 |
|  |  |  |  |  |  |  |
|  | **UHC index & GLOBOCAN ASMR** | | **5-year survival rate & GLOBOCAN ASMR** | |  | |
|  | corr | w. corr | corr | w. corr |  |  |
| removal | -0.085 | **-0.686** | -0.234 | **-0.604** |  |  |
| replace with zero |  |  | -0.049 | -0.397 |  |  |
| replace with mean |  |  | -0.209 | -0.464 |  |  |
| MICE |  |  | -0.010 | -0.268 |  |  |
|  |  |  |  |  |  |  |
